# Beyond Genetic Indicators: How Reproductive Mode and Hybridisation Challenge Freshwater Mussel Conservation

**DOI:** 10.1111/mec.70066

**Published:** 2025-08-11

**Authors:** Ellika Faust, Julie Conrads, Marco Giulio, Claudio Ciofi, Chiara Natali, Philine G. D. Feulner, Alexandra A.‐T. Weber

**Affiliations:** ^1^ Department of Aquatic Ecology Swiss Federal Institute of Aquatic Science and Technology (Eawag) Dübendorf Switzerland; ^2^ Department of Fish Ecology and Evolution Swiss Federal Institute of Aquatic Science and Technology (Eawag) Kastanienbaum Switzerland; ^3^ Department of Biology University of Florence Sesto Fiorentino (FI) Italy; ^4^ Institute of Ecology and Evolution University of Bern Bern Switzerland

## Abstract

Genetic diversity is a fundamental aspect of biodiversity, yet it is rarely assessed and monitored in conservation practice. Unionid freshwater mussels exemplify the dramatic loss of biodiversity in freshwater ecosystems, yet genomic data for these ecologically important species remain scarce. Here, we conducted a high‐resolution population genomics study of all *Anodonta* species in Switzerland, with a focus on two species with contrasting reproductive strategies. After generating draft genomes of the hermaphroditic *Anodonta cygnea* and the gonochoric *Anodonta anatina* , we performed whole‐genome resequencing of 421 individuals collected in 31 localities. While *A. anatina* populations followed a metapopulation structure shaped by catchment areas, genetic diversity correlated positively with waterbody size, suggesting greater vulnerability in small ponds compared with large lakes. Inbreeding levels were low; however, effective population sizes were consistently below 100, indicating serious extinction risks. Strong divergence between *A. anatina* populations north and south of the Alps suggests a putative undescribed *Anodonta* species in the Ticino area. Furthermore, we detected hybridisation between *A. cygnea* and *A. exulcerata* , indicating genomic permeability between these species. In addition, genomic data suggested facultative selfing in *A. cygnea* , leading to a marked reduction in genetic diversity, increased population structure and inbreeding and a decline in effective population size compared to the outcrossing *A. anatina* . Our study underscores that reproductive strategy fundamentally shapes genetic indicators of biodiversity and influences extinction risk; conservation targets should therefore be adapted to the biology of the species of interest. To conclude, we advocate for integrating reproductive mode and genomic data into conservation planning to more accurately assess vulnerability and guide effective action.

## 1 Introduction

The Anthropocene is characterised by a global biodiversity crisis, with unprecedented species extinction caused by human activities (Johnson et al. 2017). The importance of intraspecific genetic diversity has been recognised in the global biodiversity framework (GBF) of the convention on biological diversity (CBD), which acknowledges that genetic diversity is key for population persistence and adaptation to changing environments and extreme events. Endangered populations and species are typically characterised by low genetic diversity and are threatened by genetic erosion that can reduce fitness and ultimately contribute to extinction (Kardos 2021; Kardos et al. 2021). Worryingly, comparative meta‐analyses have highlighted that genetic diversity is decreasing globally (Leigh et al. 2019; Shaw et al. 2025). Hence it is imperative that conservation actions consider and integrate this fundamental level of biodiversity (O'Brien et al. 2022, 2025). Essential biodiversity variables (EBVs) are a standardised set of measurements designed to monitor biodiversity changes across time and space. The genetic composition EBVs focus on the genetic diversity within and among populations of species, specifically genetic diversity, genetic differentiation, inbreeding and effective population size (Hoban et al. 2022; Paez et al. 2022). These estimates can help to identify conservation units, assess population connectivity and biodiversity loss (Hohenlohe et al. 2021). Beyond EBVs, genomic data can also reconstruct demographic history, detect hybridisation, evaluate a population's adaptive potential (Hohenlohe et al. 2021) and uncover cryptic species (Bickford et al. 2007; Struck et al. 2018). Clear species definitions are crucial in conservation, as actions largely occur at the species level (e.g., IUCN Red List). Undescribed cryptic species risk being overlooked, potentially leading to underestimated vulnerability or unnoticed extinctions (Carmi et al. 2016).

The global decline in biodiversity is especially pronounced in freshwater ecosystems, where 85% of assessed biodiversity has been declining since the 1970s—significantly higher than the 56% and 69% observed in marine and terrestrial ecosystems, respectively (WWF 2024). This marked decline is driven by two key factors (Dudgeon et al. 2006; Sayer et al. 2025). First, freshwater habitats are inherently vulnerable due to their fragmented nature, limited connectivity and restricted dispersal via river networks. Second, freshwater ecosystems face intense anthropogenic pressure, stemming from the direct use of water resources for purposes such as wastewater and drinking water plants, dams and hydropower, fishing, irrigation, channelisation and pollution from land use. Freshwater mussels (Bivalvia: Unionida) are considered keystone species, integral to the health of these ecosystems, providing critical services such as nutrient cycling and water purification (Vaughn 2018). Globally, nearly half of the 456 species evaluated by the IUCN are classified as threatened or near‐threatened (Böhm et al. 2021). This decline is driven by several factors, including habitat destruction and modification, invasive species, pollution and climate change (Lopes‐Lima et al. 2014, 2017). Their life‐history traits make them particularly vulnerable to environmental disturbances: they exhibit high longevity, reach sexual maturity late and have a complex life cycle that includes an obligatory parasitic larval stage on a host fish (Lopes‐Lima et al. 2017). While some species are generalists regarding host fish species, others have strong host specificity, making them particularly vulnerable when host fish populations are also declining (Modesto et al. 2018). Importantly, this larval parasitic stage is the primary mechanism of mussel dispersal and colonisation of new habitats (Modesto et al. 2018).

While 25 species of freshwater mussels have been described in Europe, the true diversity of these mussels is likely underestimated due to the reliance on shell morphology for species identification, which is highly variable. This has led to historical splitting and lumping of species (Haas 1969; Graf 2010). However, recent molecular studies have shown that some splits were justified, such as the resurrection of *Anodonta exulcerata* as a distinct species from *Anodonta cygnea* (Froufe et al. 2017; Riccardi et al. 2020). Additionally, the *Unio crassus* species complex has been recently completely re‐evaluated, revealing 12 biological species (Lopes‐Lima et al. 2024). Molecular studies have also revealed that the widespread *A. anatina* likely represents a complex of cryptic species, with five divergent mitochondrial lineages and at least three evolutionary significant units, which may represent cryptic species (Froufe et al. 2014, 2017; Lopes‐Lima et al. 2016; Riccardi et al. 2020; Lyubas et al. 2023). Despite extensive mitochondrial data, few studies have analysed nuclear data, and then only a limited number of markers (Lopes‐Lima et al. 2016). Hence, there is to date no study that has used whole‐genome data for species delimitation and for the assessment of genetic EBVs in freshwater mussels.

This study focuses on Switzerland, as conservation policies, jurisdiction and biodiversity reporting are implemented at the national level. Despite its small geographic extent, the country plays a crucial role in the biogeography of freshwater biodiversity and is often referred to as the ‘water castle of Europe’. The Alps give rise to several major river catchments, including the Rhine, Rhône and Ticino, as well as key sub‐catchments such as the Limmat, Reuss and Aare, all of which ultimately flow into the Rhine. Given this complex hydrological network, strong genetic differentiation over short geographic distances is expected. In Switzerland, in addition to *A. anatina* , two other *Anodonta* species are present: *A. exulcerata* and *A. cygnea* (Pfarrer et al. 2022). While *A. anatina* is typically gonochoric (although occasional hermaphroditism has been reported (Hinzmann et al. 2013)), *A. cygnea* is known to be predominantly hermaphroditic (Lima et al. 2012), with laboratory studies reporting instances of self‐fertilisation (Bloomer 1940, 1943). However, the prevalence of self‐fertilisation in natural populations remains unclear. Despite their coexistence in Switzerland, *A. anatina* and *A. cygnea* are not closely related, with an estimated divergence time of around 25 million years (Lopes‐Lima et al. 2021, 2024). Both species are considered host generalists; however, some species‐specific preferences have been described (Huber and Geist 2017, 2019). The aim of this study was to assess genetic EBVs using high‐quality genomic data for all *Anodonta* species present in Switzerland, in alignment with the reporting requirements of the CBD. In addition to estimating EBVs, our genomic analyses revealed cryptic diversity, evidence of hybridisation and insights into genetic consequences of a shift in reproductive strategy.

## 2 Methods

### 2.1 Draft Genomes of *Anodonta cygnea* and *Anodonta anatina*

#### 2.1.1 Sampling and Molecular Species Identification

Collection permits were requested in advance from the relevant cantonal authorities of nature protection (Canton Aargau for mussels collected in Scherz; Canton Zürich for mussels collected in Türlersee). Five adult *Anodonta anatina* individuals were hand‐collected from a small stream in Scherz, Switzerland (47°26′40.20″ N 8°11′05.89″ E) on the 24th of February 2023 and brought back alive to the Eawag laboratory in Dübendorf, Switzerland. Two adult individuals of the swan mussel *A. cygnea* were collected by snorkelling in Türlersee, Switzerland (47°15′57.2″ N 8°30′35.6″ E) on the 7th of July 2023 and brought back alive to the laboratory in Dübendorf. All mussels were kept alive in the Eawag experimental facility Aquatikum until processing. They were maintained in an open‐water circuit at 19°C and fed twice a week using a mixture of lab‐grown algae. Given the morphological plasticity of *Anodonta* mussels, species identification was initially based on shell morphology and subsequently confirmed by sequencing a COI barcode. Briefly, a small tissue biopsy was taken from the foot of each of the seven individual mussels and DNA was extracted using the E.Z.N.A Tissue DNA kit (Omega Bio‐tek) according to the manufacturer's instructions. DNA integrity, quantity and purity were assessed using NanoDrop Eight (thermo scientific; Witec AG). A fragment of the COI mitochondrial gene was amplified with forward primer LCO1490‐JJ (5′–3′ primer sequence: CHACWAAYCATAAAGATATYGG) and reverse primer HCO2198‐JJ (5′–3′ primer sequence: AWACTTCVGGRTGVCCAAARAATCA). PCR cycling conditions were: an initial step of 95°C for 15 min, followed by 35 cycles of 94°C for 30 s, 51°C for 90 s, 72°C for 60 s and then a final extension of 72°C for 10 min. Amplified PCR products were purified and Sanger sequenced by Microsynth AG, Balgach, Switzerland. The COI sequences of the seven *Anodonta* individuals were compared to the NCBI GenBank database using the Basic Local Alignment Search Tool (BLAST) to confirm morphological species identification.

#### 2.1.2 High Molecular Weight DNA Extraction, Library Preparation and PacBio HiFi Sequencing

For *A. cygnea* , the two individuals were dissected on a glass plate on ice and small tissue pieces were flash frozen in liquid nitrogen. The tissue samples were then sent to the University of Florence, Italy, for high‐molecular weight (HMW) DNA extraction, library preparation and PacBio HiFi sequencing conducted as part of the European Reference Genome Atlas project Biodiversity Genomics Europe (ERGA‐BGE). Tissue leftovers from both individuals were deposited as vouchers at the Natural History Museum in Bern, Switzerland (voucher IDs: NMBE584869 and NMBE584870).

HMW DNA was extracted from the specimen NMBE584870 using the E.Z.N.A. Mollusc & Insect DNA Kit (Omega Bio‐Tek) according to the manufacturer's protocol. Nucleic acids yield was quantified in a Qubit fluorimeter using a Qubit dsDNA HS Assay (Life Technologies) and DNA purity was assessed by comparing absorbance values at 260, 280 and 230 nm in a Tecan Infinite M200 Pro spectrophotometer using a NanoQuant plate (Tecan). Integrity of DNA was then assessed by pulsed‐field gel electrophoresis. Whole DNA was sheared in a Megaruptor 2 DNA shearing system (Diagenode) using large fragment hydropores with a target mean fragment length of 20 kb. Fragments profile was assessed by capillary electrophoresis on a Fragment Analyzer using an HS large fragment 50 kb kit (Agilent Technologies). Repair and A‐tailing of DNA fragments and SMRTbell adapters ligation were performed according to the SMRTbell prep kit 3.0 protocol (Pacific Biosciences). Primer annealing, polymerase binding and preparation of internal DNA control were performed using the Pacific Biosciences Sequel II binding kit and DNA internal control complex 3.2 according to the manufacturer's protocol. Library fragments shorter than 10 kb were removed by eluting libraries in a Blue Pippin automated pulse‐field gel electrophoresis device (Sage Science). Sequencing runs were set up using SMRT Link v11.1. Samples were sequenced in HiFi mode in a Pacific Biosciences Sequel IIe platform using Sequel II Sequencing plates 2.0 and two 8 M ZMW SMRT cells with a 30‐h movie time and 2 h of pre‐extension time for a target 25× genome coverage.

For *A. anatina* , several HMW DNA extractions were performed in the laboratory at Eawag, Dübendorf. Despite apparent initial good quality and integrity, the DNA was consistently degraded when quality control was performed at the sequencing centre (next‐generation sequencing platform (NGSP) in Bern, Switzerland), which was likely due to strong DNases remaining in the DNA extractions. Therefore, a specimen (PB107) was brought alive to the NGSP where it was dissected and processed for HMW DNA extraction. Despite multiple tests from different protocols and kits, a satisfactory HMW DNA extraction could not be achieved. Eventually, HMW DNA was extracted using MagAttract HMW DNA Kit (Qiagen) from foot and mantle tissues. There was sufficient DNA to construct two PacBio libraries from foot and mantle tissues, respectively. Prior to SMRTbell library preparation, genomic DNA was assessed for quantity, quality and purity using a Qubit 4.0 flurometer (Qubit dsDNA HS or BR Assay kit; Q32851/Q32850, Thermo Fisher Scientific), an Advanced Analytical FEMTO Pulse instrument (Genomic DNA 165 kb Kit; FP‐1002‐0275, Agilent) and a Denovix DS‐11 UV–Vis spectrophotometer, respectively. SMRTbell libraries were prepared according to the PacBio guideline SMRTbell prep kit 3.0. Briefly, sheared gDNA was concentrated and cleaned using 1× SMRTbell clean‐up beads. The samples were then quantified and qualified to be in the range of 9–20 Kb using a Qubit 4.0 flurometer (Qubit dsDNA HS Assay kit; Q32851, Thermo Fisher Scientific) and an Advanced Analytical FEMTO Pulse instrument (Genomic DNA 165 kb Kit; FP‐1002‐0275, Agilent), respectively. The rest of the procedure as referenced above was followed including end‐repair and A‐tailing, ligation of barcoded overhang adapters and then purification of the library using AMPure PB beads as well as a nuclease treatment. The only deviation was that for libraries with ≥ 1 μg, a gel‐based size selection was employed to remove fragments below 10 Kb according to the document entitled: ‘Technical note GEL CASSETTE SIZE SELECTION METHODS FOR WGS HIFI LIBRARIES’, following the protocol for a Sage Science BluePippin device (PacBio part number 102‐326‐503). Final libraries were checked for quantity and quality using a Qubit 4.0 flurometer (Qubit dsDNA HS Assay kit; Q32851, Thermo Fisher Scientific) and an Advanced Analytical FEMTO Pulse instrument (Genomic DNA 165 kb Kit; FP‐1002‐0275, Agilent). Instructions in SMRT Link Sample Setup were followed to prepare the SMRTbell library for sequencing (PacBio SMRT Link v12). Shortly, PacBio Sequencing primer v3.2 and Sequel DNA Polymerase 3.0 were annealed and bound, respectively, to the DNA template libraries using a Sequel II Binding Kit 3.2 (PacBio Part number 102‐333‐300) and the complex was cleaned using SMRTbell clean‐up beads. Both libraries were loaded at an on‐plate concentration of 120–170 pM using adaptive loading, along with the use of Sequel II DNA internal control complex. SMRT sequencing was performed in CCS mode on the Sequel IIe with Sequel Sequencing kit 3.0, SMRT Cells 8 M, a 2‐h pre‐extension followed by a 30‐h movie time and via PacBio SMRT Link v12. Thereafter, the CCS generation is performed on the Sequel IIe and the read segmentation workflow was run in SMRT Link v12. All steps were performed at the next‐generation sequencing platform, University of Bern, Switzerland.

#### 2.1.3 Genome Assembly

To assess *A. cygnea* genome features, a 21 bp k‐mer database was generated with HiFi data (QV ≥ 20) from both cells using the count function of meryl v1.3 (Rhie et al. 2020). K‐mer frequencies and genome statistics were obtained using GenomeScope v2.0 (Ranallo‐Benavidez et al. 2020). Genome assembly was then conducted using hifiasm v0.19.9 (Cheng et al. 2021), using default parameters and the purging parameter −l set to 1, given the low level of estimated genome‐wide heterozygosity (0.34%). Assembly metrics were retrieved using asmstats, and assembly completeness was assessed with BUSCO v5.2.2 (Simão et al. 2015). The Mollusca OrthoDB database (odb10; 2024‐01‐08; *n*: 5295) and the metazoa OrthoDB database (odb10; 2024‐01‐08; *n*: 954) were used for the analyses.

For the *A. anatina* draft genome, a 31 bp k‐mer database was generated from HiFi reads (QV ≥ 20) obtained from two separate libraries (foot and mantle tissue) using the count function of meryl v1.3. The two k‐mer databases were merged with the union–sum function of meryl. K‐mer frequencies and genome statistics were obtained using GenomeScope v2.0. Genome assembly was then conducted using hifiasm v0.16.1‐r375, using default parameters and the purging parameter −l set to 3. Assembly metrics were retrieved using asmstats and assembly completeness was assessed using BUSCO v5.2.2. The mollusca OrthoDB database (odb10; 2020‐08‐05; *n*: 5295) and the eukaryota OrthoDB database (odb10; 2020‐09‐10; *n*: 255) were used for the analyses.

### 2.2 Population Genomics of *Anodonta* Mussels

Collection permits for population sampling were obtained in advance from the relevant cantonal authorities of nature protection. *Anodonta* mussels were sampled across 31 sampling sites in Switzerland from May to September 2023 (Table 1). The sites were selected based on mussel observations reported in the Swiss Center for the Cartography of the Fauna (www.infofauna.ch), with the goal of covering all six major river catchments and sub‐catchments: Aare, Limmat, Reuss, Rhine, Rhône and Ticino. In total, 421 *Anodonta* specimens were collected by wading, snorkelling or SCUBA diving, with a maximum of 25 individuals per location (Table 1; Table S1). After morphological examination, a small foot biopsy was sampled non‐lethally and immediately preserved in 100% ethanol. After tissue sampling, all mussels were placed back in their original sampling locations. Due to the morphological overlap among *Anodonta* species commonly occurring in sympatry, molecular species identification was performed using COI barcoding as described above. DNA extracted from each individual was used for individual library preparation with the PCR‐free DNA Prep Kit (Illumina). All libraries were sequenced with 150 bp paired‐end reads on a single 25B flow cell using a Novaseq X Plus sequencer at the Functional Genomics Center Zürich, Switzerland.

**TABLE 1:** Sampling locations, dates and number of individual mussels collected during fieldwork.

Catchment	Location	GPS coordinates	Collection date	*Anodonta anatina*	*Anodonta* sp.	*Anodonta cygnea*	*Anodonta exulcerata*	# Mussels
Aare	Baldeggersee	47.18734, 8.27593	2023‐06‐15	2		8		10
	Brümeliweiher	47.24881, 7.74386	2023‐06‐06	16				16
	Burtignière	46.56175, 6.17020	2023‐05‐20	11				11
	Nottwil	47.13924, 8.13897	2023‐06‐22	15				15
	Scherz	47.44438, 8.18497	2023‐02‐24	10				10
	St. Blaise	47.01104, 6.98064	2023‐07‐06	4		11		15
	Villnachern	47.47765, 8.19166	2023‐06‐06			14		14
Limmat	Lachen	47.19864, 8.85223	2023‐06‐16	10				10
	Schmerikon	47.22378, 8.94609	2023‐06‐16	12				12
	Türlersee	47.26588, 8.50990	2023‐06‐08	19		8		27
	Wädenswil	47.24129, 8.66017	2023‐06‐30			1		1
	Walensee Gäsi	47.12846, 9.10931	2023‐08‐22	14				14
	Walenstadt	47.11724, 9.29911	2023‐08‐22	14		1		15
Reuss	Ägerisee	47.13331, 8.61055	2023‐06‐09	16				16
	Alte Reuss	47.36925, 8.33102	2023‐06‐22	4		6		10
	Rotsee	47.07356, 8.32400	2023‐06‐20	6		12		18
	Sarnersee	46.84574, 8.20254	2023‐06‐20	1		10		11
	Seewen	47.03004, 8.62123	2023‐06‐27	10		5		15
	Ufschötti	47.04540, 8.32192	2023‐08‐12			20		20
Rhein	Alter Rhein	47.37631, 9.66837	2023‐06‐21	13		2		15
	Bildweiher	47.40595, 9.30892	2023‐06‐07	3				3
	Eichweiher	47.42280, 9.39268	2023‐08‐11	8		8		16
	Greifensee	47.37289, 8.65891	2023‐08‐23	7		4		11
	Nussbaumersee	47.61362, 8.81382	2023‐08‐09			1		1
	Rütiweiher	47.48840, 9.26817	2023‐08‐11	2		9		11
	Vagoweiher	47.58635, 9.02926	2023‐08‐09	25				25
Rhone	Etang du Duzillet	46.28875, 6.96066	2023‐08‐18			12		12
	Etang du Rosel	46.12903, 7.05919	2023‐08‐15			16		16
	Lac de Bret	46.51504, 6.77159	2023‐08‐16	21				21
Ticino	Agno	45.99188, 8.90132	2023‐08‐08			3	16	19
	Locarno	46.16012, 8.80529	2023‐08‐08		10		1	11
Total				243	10	151	17	421

#### 2.2.1 Data Processing

Following an initial quality check with FastQC 0.12.1 (Andrews 2010), sequencing reads from all individuals were first mapped to the *A. anatina* genome assembly using BWA‐MEM2 (Vasimuddin et al. 2019) to analyse all species together (Dataset 1: all species). Reads with a mapping quality score below 20 were removed using Sambamba 1.0.1 (Tarasov et al. 2015), and optical/PCR duplicates were filtered out using Picard MarkDuplicates 3.3.0 (https://broadinstitute.github.io/picard/). Genotype calling was performed with bcftools 1.20 (Danecek et al. 2011, 2021), followed by filtering with bcftools, vcftools 0.1.16 (Danecek et al. 2011) and plink PLINK 1.9 (Purcell et al. 2007) (see Table S2 for filtering parameters). Linkage disequilibrium (LD) pruning was performed using PCAone (Bercovich et al. 2025) in 100 kb windows with an r^2^ threshold of 0.1 and *k* = 3. PCAone calculates an adjusted measure of LD by using the top inferred principal components to correct for population structure and admixture.

To improve genomic inference, resequencing data were mapped to the least divergent or species‐specific reference genome, resulting in two datasets ( *A. anatina* and *A. cygnea* + *A. exulcerata* ). Mussels were first classified as *A. anatina* , *A. cygnea* or *A. exulcerata* based on their COI barcode. This classification determined which sequences were mapped to the *A. anatina* or *A. cygnea* genome. For *A. exulcerata* individuals, the genome of *A. cygnea* —its closest sister species—was used for mapping. Sequences were processed the same way as for Dataset 1 described above (Table S2). After filtering, the average sequencing depth ranged from 2.6× to 8.8× for the *A. anatina* dataset, while it ranged from 2.5× to 10.4× for the *A. cygnea* + *A. exulcerata* dataset. The final three filtered datasets contained the following number of single‐nucleotide polymorphisms (SNPs): all species—400 individuals and 26,704,704 SNPs; *A. anatina*—243 individuals and 27,996,116 SNPs; and the *A. cygnea* + *A. exulcerata* dataset—155 individuals and 22,403,827 SNPs.

#### 2.2.2 Data Analyses

PCA was performed on the LD‐filtered data in PLINK 1.9 (Purcell et al. 2007). Admixture proportions were estimated using ADMIXTURE 1.3 (Alexander et al. 2009). For the admixture analysis of *A. anatina* , the LD‐pruned data were downsampled to 2 million SNPs for computational efficiency. Pairwise *F*_ST_ values were computed in PLINK 2 (Chang et al. 2015) using the Hudson method (Hudson et al. 1992; Bhatia et al. 2013), excluding locations with fewer than five individuals to ensure statistical robustness. Isolation by distance (IBD) was assessed among samples in the Rhine and its sub‐catchments by correlating genetic distance (*F*_ST_/1−*F*_ST_) with geographic distance (shortest geographic distance along the Swiss river network (https://www.swisstopo.admin.ch/de/landschaftsmodell‐swisstlmregio, Federal Office of Topography swisstopo)). Significance was tested using a Mantel test. Pairwise kinship coefficients were estimated using the KING algorithm as implemented in PLINK 2. To provide a measure of genetic dissimilarity between pairs of individuals among all species, distances were also generated based on identity‐by‐state with PLINK 1.9. Before phylogenetic maximum likelihood inference, the LD‐pruned data were filtered to retain only SNPs with at least one homozygous individual for the reference and the alternate alleles and was then converted to a PHYLIP file using vcf2phylip v.2 (Ortiz 2019). Phylogenetic relationships were inferred using RAxML‐NG 1.2.2 (Kozlov et al. 2019) using the GTR + ASC_LEWIS substitution model.

Genetic diversity was evaluated with nucleotide diversity (π), observed and expected heterozygosity (*H*o, *H*e) using vcftools. Nucleotide diversity was estimated in 10 kb windows for locations with five or more individuals. Heterozygosity estimates were normalised by dividing heterozygote counts by the total genome size. Inbreeding was assessed by estimating the inbreeding coefficients (*F*_IS_) with vcftools for locations with five or more individuals and runs of homozygosity (ROH). Individual ROH were calculated using the full dataset with the hidden Markov model approach in bcftools. Allele frequencies were calculated from each species and only called genotypes were used for ROH estimation. Individual inbreeding coefficients (FRoH) were calculated by dividing the total length of ROH > 300 kb by the total genome size. Finally, contemporary effective population size (*N*e) was estimated with currentNe (Santiago et al. 2024). CurrentNe uses chromosome numbers to correct for non‐independence among SNPs and estimates the number of full siblings in the dataset, improving the accuracy of *N*e estimates in species with complex mating systems. The full dataset was downsampled to include just under 2 million SNPs, which is the limit of currentNe. *N*e was estimated only for localities with at least 10 individuals. The genetic indicators (π, *H*o, *F*_ST_, FRoH, *F*_IS_, *N*e) were tested for correlation with the size of the sampled waterbody using Spearman's or Pearson's correlation coefficient, depending on normality. The average values of the genetic indicators and kinship across localities were compared between *A. anatina* and *A. cygnea* using Wilcoxon rank‐sum tests. Unless otherwise stated, all data manipulation and visualisation of results were performed in R v4.4.2 (Wickham et al. 2019) using Tidyverse packages (R Core Team 2024) and Rstudio 2024.09.1 + 394 (Posit Team 2024). ChatGPT‐4o was used to assist in debugging R and Bash scripts, and to refine scientific English after initial drafting by the authors.

## 3 Results

### 3.1 High‐Quality Draft Genome of *A. cygnea* and Fragmented Draft Genome of *A. anatina*

Two PacBio libraries from the same *A. cygnea* individual were constructed and sequenced on two SMRT cells, yielding a total of ~50.4 Gb of HiFi data. The HiFi reads had a mean length of 15,593 bp and 15,916 bp for the two libraries, indicating optimal read quality and fragment length. GenomeScope analysis estimated a genome‐wide heterozygosity level of 0.34% (Table S3). The assembled draft genome was of high quality, with a total size of ~2.46 Gb distributed across 2008 scaffolds, with an N50 of 2.7 Mb (Table S3). BUSCO completeness scores for Mollusca were relatively low (C: 83.5%); however, the completeness score for the broader Metazoa database was substantially higher (C: 96%), similar to other high‐quality published Unionid genomes (Table S3).

Despite numerous attempts, only two PacBio libraries of *A. anatina* could be successfully constructed and sequenced on a single SMRT cell, resulting in a limited amount of sequencing data available for this species (a total of ~26.5 Gb of HiFi data). With a mean length of 7019 bp and 6954 bp for both libraries, HiFi reads were relatively short. Given the low genome coverage (~10.6×) and the short read length, the assembly statistics were not of high quality (Table S3). Consequently, the GenomeScope model fit was poor, producing a very low genome size estimate (552–566 Mb) and a very high heterozygosity (4.1%–7.4%). The primary HiFi assembly was highly fragmented, consisting of nearly 34,000 contigs with a total length of ~2.8 Gb and BUSCO scores were low (C: 73.6% for Mollusca) (Table S3). Nevertheless, due to the relatively high genetic divergence between *A. anatina* and *A. cygnea* (Lopes‐Lima et al. 2021, 2024) and the challenges associated with using a distant reference genome for population parameter inference (Thorburn et al. 2023; Maurstad et al. 2024), we opted to use the *A. anatina* draft genome for estimating genetic EBVs for *A. anatina* . While the reference genome is fragmented, this is expected to have only a minor impact on genetic EBV estimates, with the possible exception of FRoH (see below).

### 3.2 Whole‐Genome Sequencing Suggests an Undescribed *Anodonta* Species South of the Alps

**FIGURE 1:**
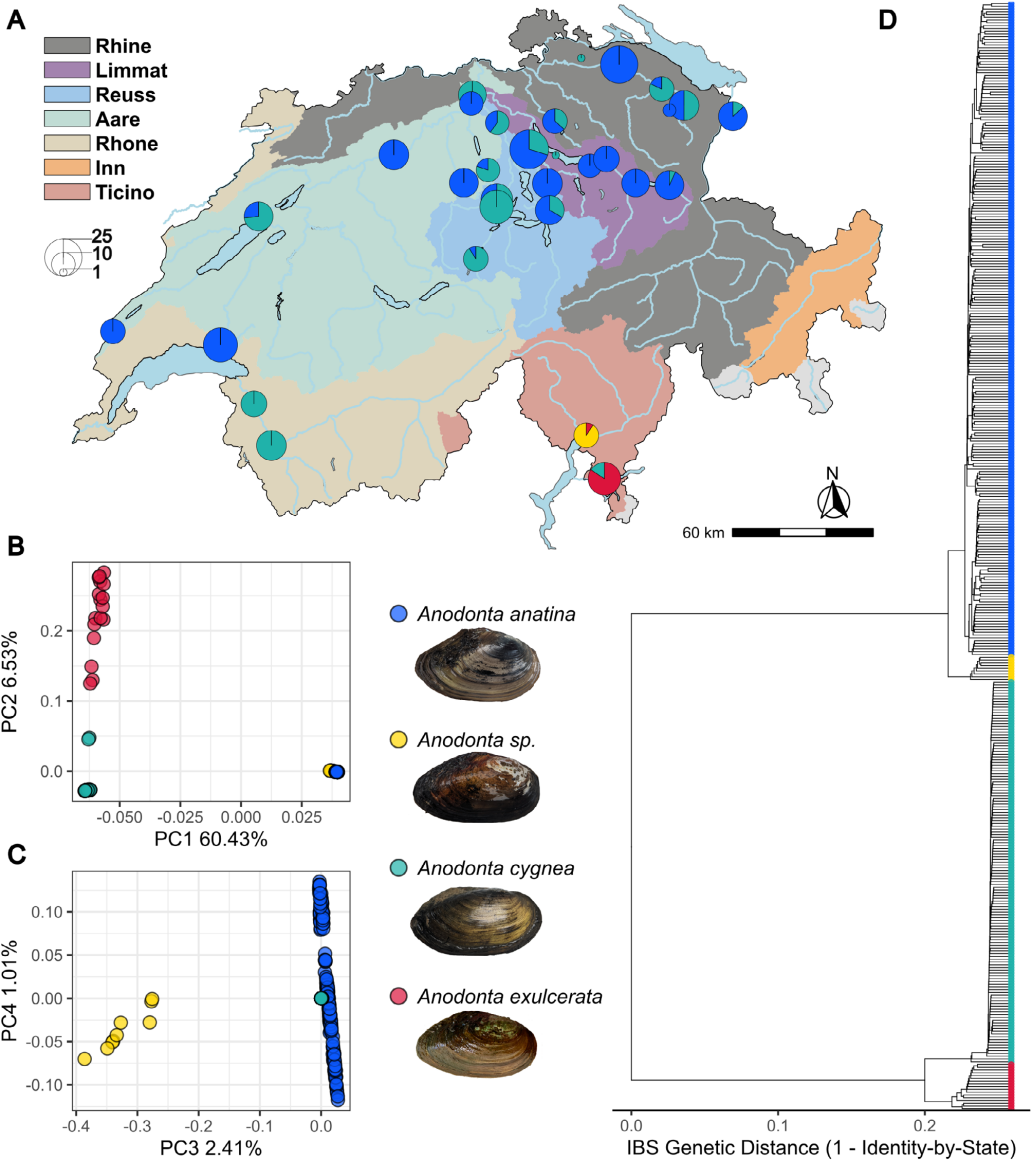
Geographic distribution and genomic divergence of *Anodonta* freshwater mussels in Switzerland. (A) Sampling sites and the number of individuals collected in this study. (B) Genomic principal components analysis (PCA), showing the first two principal components (PCs) across the four *Anodonta* lineages. PC1 distinguishes *A. cygnea* and *A. exulcerata* from *A. anatina* and *Anodonta* sp. (C) Third and fourth principal components of the genomic PCA, with PC3 separating the putative undescribed species *Anodonta* sp. from *A. cygnea* , *A. exulcerata* and *A. anatina* . (D) Genomic divergence among the four lineages, inferred from identity‐by‐state, providing a measure of genetic dissimilarity between pairs of individuals.

**FIGURE 2:**
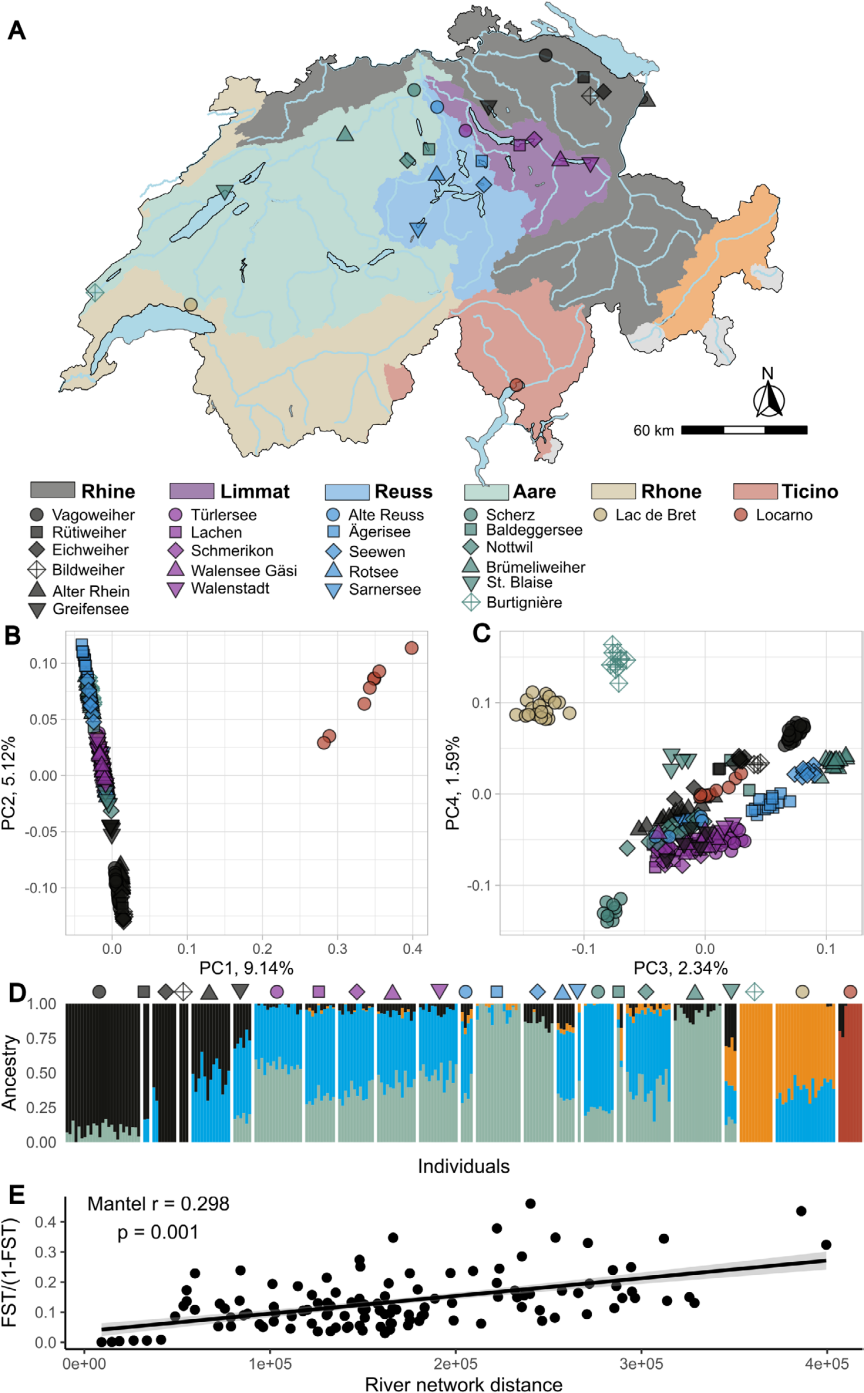
Population structure of *Anodonta anatina and Anodonta* sp. in Switzerland. (A) Sampling sites with colours corresponding to the respective river catchments. Symbols represent individual locations within each catchment. (B) Genomic principal components analysis (PCA), showing the first two principal components (PCs) across *A. anatina* and *Anodonta* sp. populations. PC1 differentiates *A. anatina* from *Anodonta* sp. which are all clustered on the right side of the panel, distinct from *A. anatina* . (C) Third and fourth principal components of the genomic PCA, further distinguishing *A. anatina* populations. (D) Clustering analysis using ADMIXTURE, identifying five genetic clusters. Symbols above the plot correspond to locations in panel A. (E) Significant isolation‐by‐distance among *A. anatina* populations connected by water.

Following sampling, we successfully sequenced 421 *Anodonta* specimens collected from 31 locations across Switzerland and assigned them to species based on COI barcodes: 253 *A. anatina* , 151 *A. cygnea* and 17 *A. exulcerata* (Table 1; Figure 1A). Specimens were collected from a diverse range of habitats, including large and small lakes, ponds, a river and a stream, highlighting the broad ecological niches of these species. *A. anatina* was the most abundant species, but *A. cygnea* frequently co‐occurred with it at several localities (Figure 1A). *Anodonta exulcerata* was restricted to Ticino, the region of Switzerland located south of the Alps. Phylogenetic analysis revealed a deep divergence between *A. anatina* and a clade comprising the closely related species *A. cygnea* and *A. exulcerata* (Figure 1D). This pattern was further supported by PCA, in which over 60% of the total genetic variation separated these two groups (Figure 1B). Within the *A. anatina* group, we identified a highly divergent population from Locarno (Ticino) (Figure 2A), which was genetically distinct from all other *A. anatina* individuals (Figures 1 and 2). This population formed a separate cluster in the PCA (Figures 1C and 2B). In the nuclear phylogenetic tree, the Locarno population appeared as a monophyletic lineage, exhibiting a level of genetic divergence from other *A. anatina* populations only slightly lower than between *A. cygnea* and *A. exulcerata* (Figure 1D). In addition, *F*_ST_ values between *Anodonta* sp. and *A. anatina* populations ranged from 0.22 to 0.36 (Figure S4). Further phylogenetic analysis of the COI gene, incorporating *A. anatina* sequences from the literature (Table S4) (Froufe et al. 2017), showed that eight COI sequences clustered with the Italian+Ebro clade, while two sequences clustered with the European clade (Figure S1). Given this mitochondrial signal and the substantial nuclear divergence, we propose that the Locarno population (Italian + Ebro clade) may represent an undescribed *Anodonta* species. Therefore, we refer to this putative new species as *Anodonta* sp. throughout the remainder of this manuscript. Interestingly, two *Anodonta* sp. individuals carried an *A. anatina* haplotype, one of these individuals having about 25% of its genome assigned to *A. anatina* (Figure 2D). Given that retention of ancestral polymorphism would likely affect more individuals,, this signal suggests recent hybridisation between *Anodonta* sp. and *A. anatina* .

### 3.3 Population Structure Follows River Catchment in *A. anatina*

**FIGURE 3:**
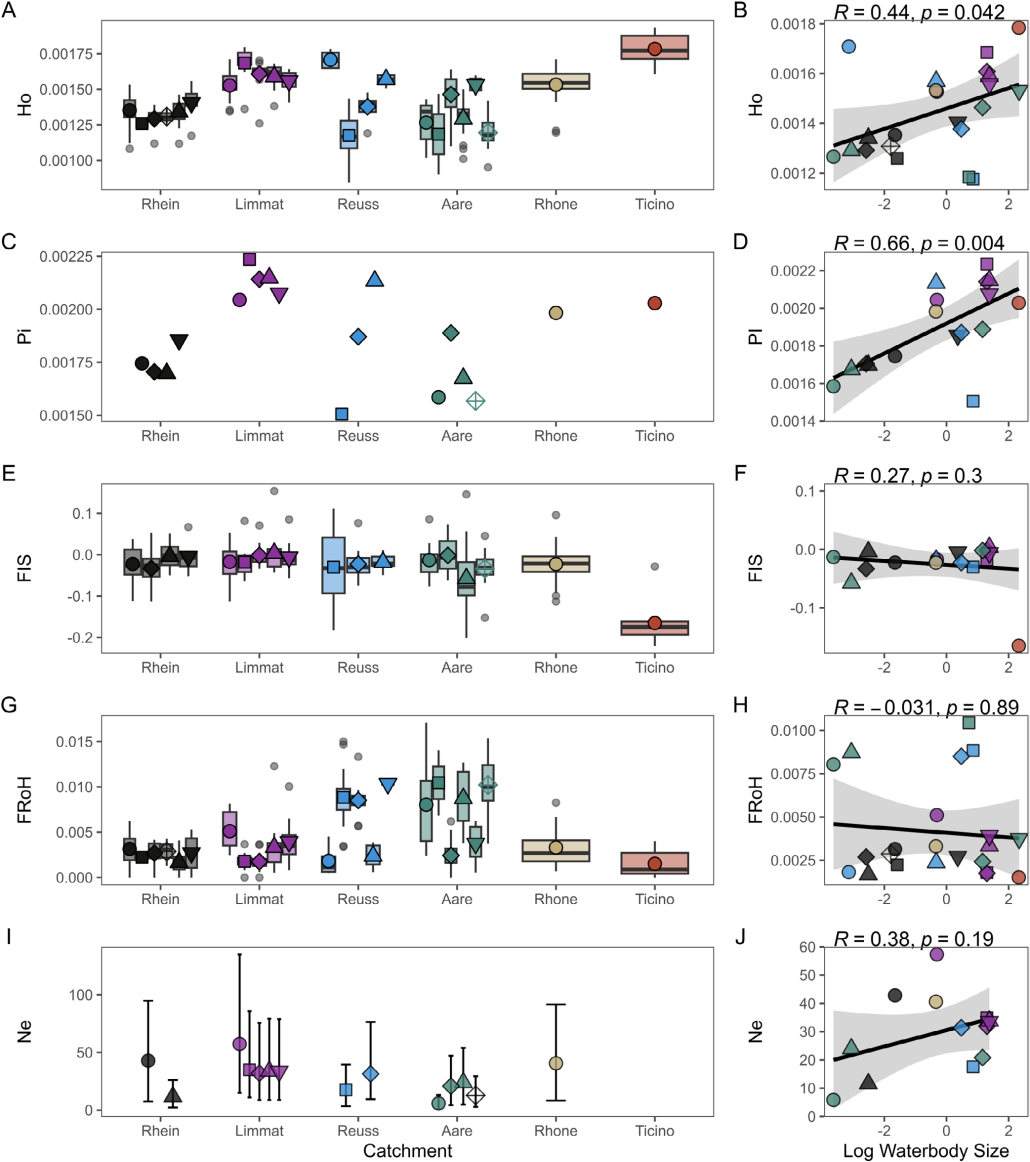
Genetic essential biodiversity variables in *Anodonta anatina and Anodonta* sp. Colours correspond to the respective river catchments and symbols correspond to sampling locations within each catchment. For a complete legend of catchment colours and location symbols, see Figure 2. Points represent sample means, and boxplots indicate the distribution of values across samples (A) Individual observed heterozygosity per sampling locality (all localities included). (B) Observed heterozygosity is positively correlated with waterbody size. (C) Genome‐wide nucleotide diversity (mean over 10 kb windows) per sampling locality, including only sites with at least five individuals. (D) Nucleotide diversity is positively correlated with waterbody size. (E) Inbreeding coefficient (*F*_IS_) per sampling locality, including only sites with at least five individuals. (F) *F*_IS_ is not correlated with waterbody size. (G) Individual inbreeding, quantified as the fraction of runs of homozygosity (FRoH), per sampling locality (all localities included). (H) FRoH is not correlated with waterbody size. (I) Effective population size (*N*e) per sampling locality, including only sites with at least ten individuals. (J) *N*e is not positively correlated with waterbody size.

Admixture analysis of *A. anatina* identified five well‐supported genetic clusters, including the *Anodonta* sp. population from Locarno (Figure 2D, Figure S2). These groups largely corresponded to major catchment areas: Ticino, Rhône and Rhine, while populations from the Limmat, Reuss and Aare catchments were more closely related, reflecting their geographic proximity (Figure S3). Notably, most populations were clustered by sampling locality, indicating significant genetic structure across populations (Table S5). Most of the populations were genetically distinct, with pairwise *F*_ST_ values ranging from 0.0008 to 0.31, excluding comparisons with *Anodonta* sp. from Ticino (which ranged from 0.22 to 0.35) (Table S5; Figures S4–S6). Two populations of the Aare catchment were particularly divergent (Figure 2C): (1) Burtignière, the only river population sampled in this study, which was also geographically distant from other populations in the Aare catchment, and (2) Scherz, a very small stream population. Due to their low diversity (Figure 3C), small effective population size (Figure 3I) and relative isolation, both populations likely experienced increased genetic drift, leading to their divergence. Furthermore, we detected significant isolation by distance, with geographically distant populations also showing greater genomic differentiation (Figure 2E). Finally, kinship estimates among populations were very low, indicating that most of the individuals among localities were not related (Table S6).

### 3.4 Genetic Diversity Is Correlated With Waterbody Size in *A. anatina*

We next investigated genetic diversity (heterozygosity and nucleotide diversity), inbreeding and effective population sizes across all *A. anatina* populations (Figure 3). Observed heterozygosity varied substantially among populations, mean *H*o ranging from 0.0012 to 0.0018 (Figure 3A). Similarly, nucleotide diversity, calculated as the genome‐wide average in 10 kb windows, ranged from 0.0015 to 0.0022 (Figure 3C). Notably, both measures of genetic diversity were positively correlated with waterbody size, indicating that populations in large lakes exhibited higher genetic diversity compared to those in small ponds (Figure 3B,D). The inbreeding coefficient (*F*_IS_)—which measures the deficit or excess of heterozygotes relative to Hardy–Weinberg expectations—was generally close to zero, suggesting no strong deviations from Hardy–Weinberg equilibrium (Figure 3E,F). The exception was the *Anodonta* sp. population from Locarno (Ticino), which exhibited a negative *F*_IS_, indicating an excess of heterozygotes. However, given that only one population was analysed, and that the use of a heterospecific reference genome can introduce a bias towards excess heterozygosity (Maurstad et al. 2024), this result should be interpreted with caution and is not explored further here. To assess individual inbreeding more directly, we estimated FRoH, the proportion of the genome in long runs of homozygosity (ROH) (Figure 3G,H, Figure S7). While some variation was observed among populations, individual inbreeding appeared to be minimal, with only small ROH and FRoH values below 0.018. However, these estimates should be interpreted cautiously, as the fragmented nature of the *A. anatina* draft genome likely limits the detection of very long ROH. Finally, we estimated effective population size (*N*e), restricting calculations to locations with at least 10 sampled individuals to minimise bias. Estimates were uniformly low, ranging from 5 to 57 (Figure 3I). Contrary to genetic diversity, *N*e was not significantly correlated with waterbody size, although this may reflect the limited number of populations analysed (Figure 3J). Finally, average kinship estimates among individuals within each population were mostly low, ranging from 0.03 to 0.27 (Figure S8).

### 3.5 Ongoing Hybridisation Between *A. cygnea* and *A. exulcerata*

**FIGURE 4:**
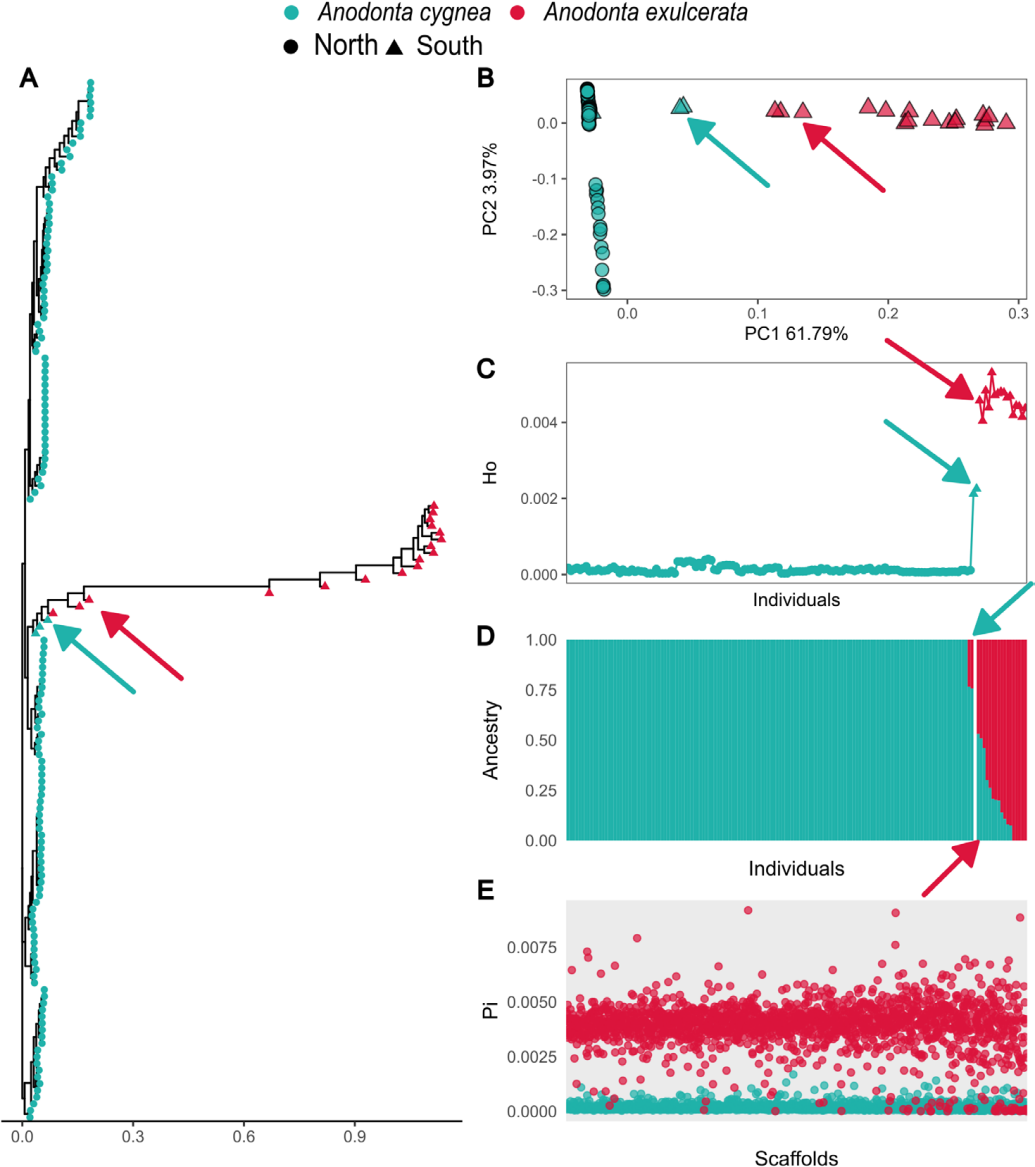
Hybridisation between *Anodonta cygnea* and *Anodonta exulcerata* . (A) Maximum‐likelihood phylogenetic reconstruction highlights hybrid individuals ‘bridging’ *A. cygnea* and *A. exulcerata* (indicated by arrows, same individuals in further panels). Sample colours denote species identity, while symbols differentiate individuals originating from either north or south of the Alps. (B) Genomic principal components analysis (PCA) reveals intermediate individuals between *A. cygnea* and *A. exulcerata* along PC1. (C) Individual observed heterozygosity across all *A. cygnea* and *A. exulcerata* individuals. Hybrid *A. cygnea* individuals exhibit intermediate heterozygosity levels between the two species. (D) ADMIXTURE analysis shows a significant proportion of the *A. cygnea* genome in most *A. exulcerata* individuals. (E) Genome‐wide nucleotide diversity (mean over 10 kb windows) per scaffold for each species. Nucleotide diversity differs markedly between species.

We identified intriguing patterns in the PCA, where some individuals were positioned between the *A. cygnea* and *A. exulcerata* clusters (Figure 4B, Figure S9). This suggests ongoing hybridisation in Agno (Ticino), the only locality of our dataset where both species occur in sympatry (Figure 1A). Although only three *A. cygnea* individuals were found at this site, their genetic patterns were notable. One *A. cygnea* individual clustered with *A. cygnea* populations from north of the Alps, whereas the remaining two *A. cygnea* individuals, along with three *A. exulcerata* individuals—each assigned to species by COI barcoding—appeared admixed (Figure 4A,B). Further evidence of admixture was detected in the ancestry analysis, where most *A. exulcerata* individuals displayed a genomic background partially shared with *A. cygnea* (Figure 4D, Figure S10). Notably, the two admixed *A. cygnea* individuals displayed particularly high levels of observed heterozygosity compared to the rest of the *A. cygnea* population (Figure 4C). Additionally, we detected elevated nucleotide diversity in *A. exulcerata* (Figure 4E), exceeding not only that of *A. cygnea* but also surpassing diversity levels found in *A. anatina* (Figure 3C).

### 3.6 Genomic Data Suggest Facultative Selfing in *A. cygnea*

**FIGURE 5:**
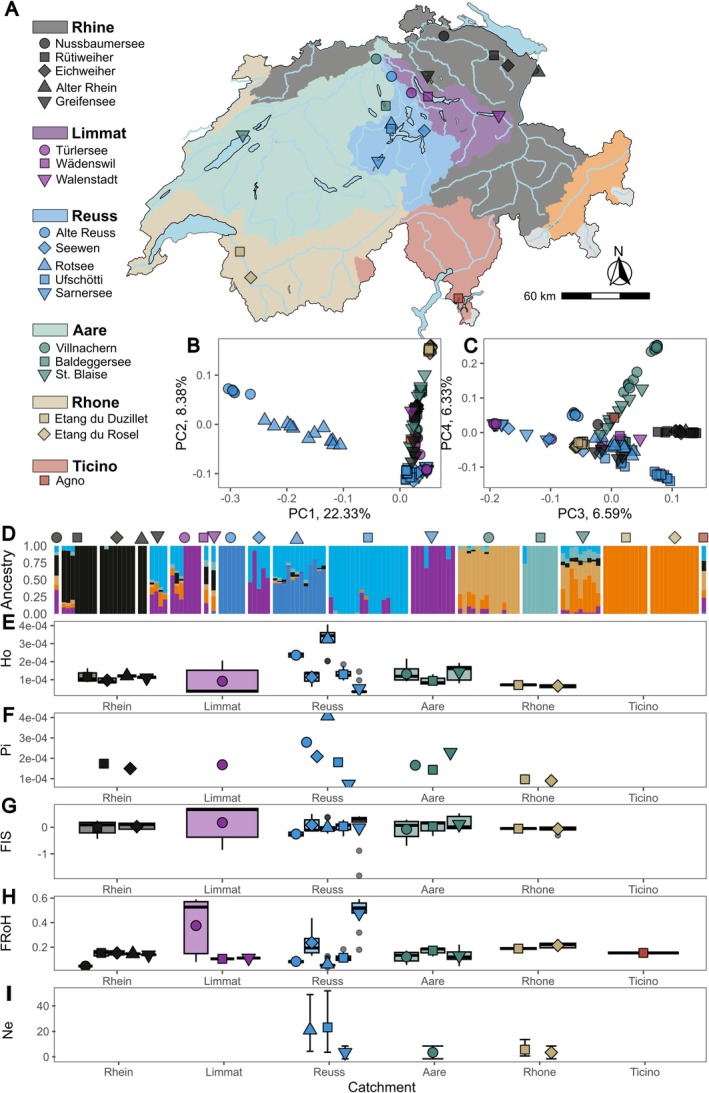
Genetic essential biodiversity variables in *Anodonta cygnea* . (A) Sampling sites of *A. cygnea* with colours corresponding to the respective river catchments. Symbols represent individual locations within each catchment. Points represent sample means, and boxplots indicate the distribution of values across samples. (B) Genomic principal components analysis (PCA), showing the first two principal components (PCs) across *A. cygnea* populations. (C) Third and fourth principal components of the genomic PCA, further distinguishing *A. cygnea* populations. (D) Clustering analysis using ADMIXTURE, identifying seven genetic clusters. Population codes correspond to panel A. (E) Individual observed heterozygosity per sampling locality (all localities included). (F) Genome‐wide nucleotide diversity (mean over 10 kb windows) per sampling locality, including only sites with at least five individuals. (G) Inbreeding coefficient (*F*_IS_) per sampling locality, including only sites with at least five individuals. (H) Individual inbreeding, quantified as the fraction of runs of homozygosity (FRoH), per sampling locality (all localities included). (I) Effective population size (*N*e) per sampling locality, including only sites with at least ten individuals.

We next examined the population structure of *A. cygnea* in greater detail (Figure 5A), excluding the two hybrids (Figure 5B,C). Genetic structure was highly pronounced, as the admixture analysis identified seven well‐supported genetic clusters (Figure 5D, Figure S11). Additionally, two populations (Alte Reuss and Rotsee) were particularly divergent, together accounting for about 22% of the genetic variation in the first axis of the genomic PCA (Figure 5B). Further principal component axes also differentiated populations from one another (Figure 5C, Figure S12). The strong genetic structuring was especially evident in the phylogenetic tree, where all individuals clustered within their respective populations (Figure S13), and in the high genetic differentiation among populations, with *F*_ST_ values ranging from 0.04 to 0.68 (Table S7, Figure S14). An intriguing finding emerged from the kinship estimates within populations. Under this framework, an individual's kinship coefficient with itself is 0.5, while full siblings have a coefficient of 0.25. In many populations, the average kinship coefficient exceeded 0.1, indicating that individuals were highly related (Figure S15, Table S8). Additionally, some individuals from different localities were nearly genetically identical, indicating a reproductive mode other than strict outcrossing (Figure S13). Furthermore, genetic diversity levels, both in terms of heterozygosity and nucleotide diversity, were particularly low (Figure 5E,F). While the inbreeding coefficients were not high (Figure 5G), we observed exceptionally high levels of individual inbreeding, with FRoH values ranging from 0.3 to 0.59 (Figure 5H). Taken together, these results suggest that *A. cygnea* likely reproduces via facultative selfing. The term ‘facultative’ is appropriate because we observed hybrids with *A. exulcerata* , indicating that *A. cygnea* retains the capacity for outcrossing. Additionally, some genetic difference persisted within populations, which would not be expected in an obligate selfer (Figure S13). In line with these observations, effective population size (*N*e) estimates were very low, ranging from 4 to 24 (Figure 5I). Finally, none of the genetic indicators were correlated with waterbody size, except for *F*_IS_ (Figure S16) and there was no evidence for isolation by distance (Figure S17).

### 3.7 Reproductive Mode Significantly Influences Genetic Indicators

**FIGURE 6:**
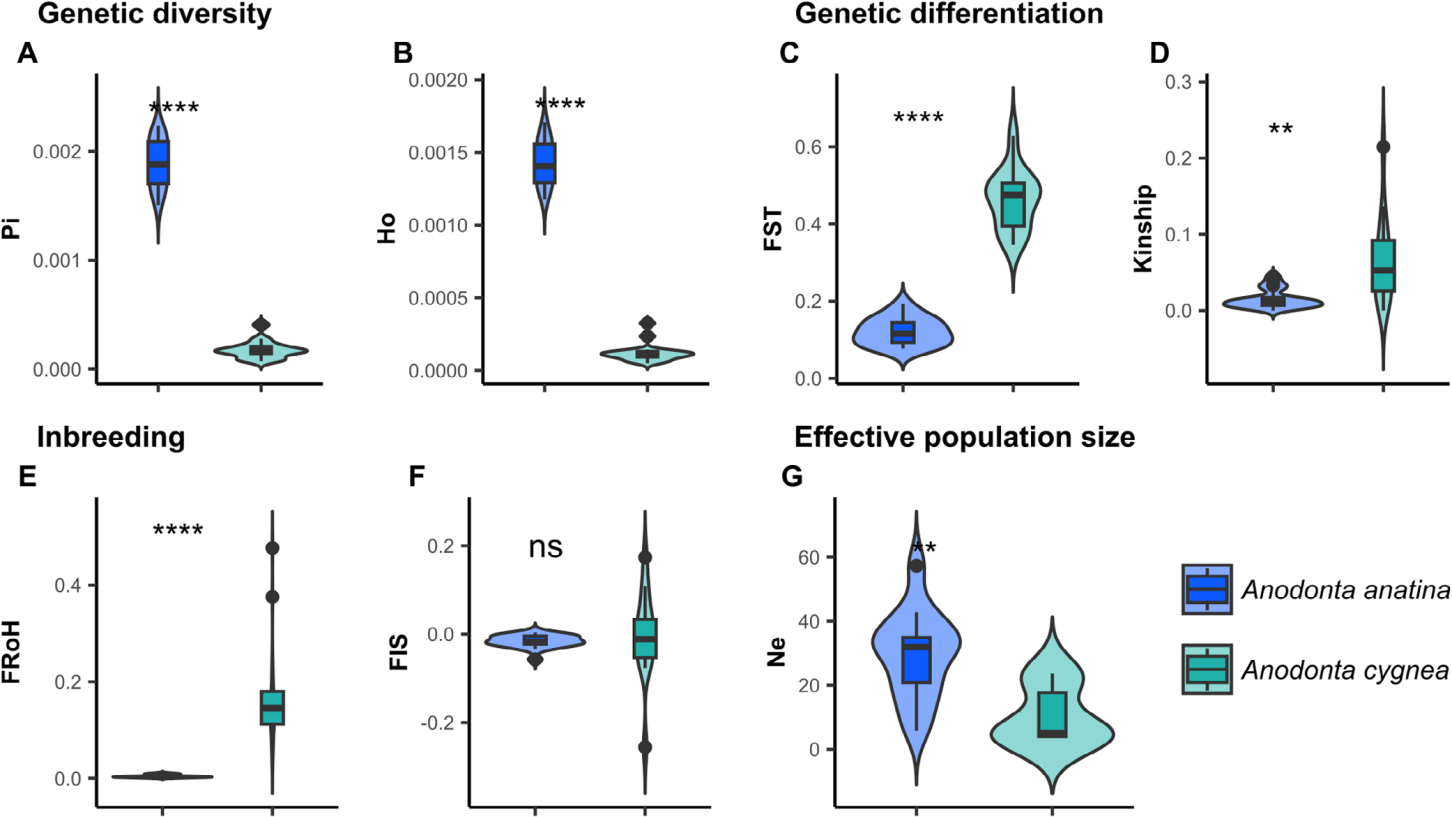
Comparison of genetic indicators between *Anodonta anatina* and *Anodonta cygnea* . (A) Nucleotide diversity is significantly higher in *A. anatina* (calculated for sites with at least five individuals; Wilcoxon rank‐sum test: *P* < 0.0001). (B) Observed heterozygosity is significantly higher in *A. anatina* (calculated for all sites with *n* > 1; Wilcoxon rank‐sum test: *P* < 0.0001). (C) Genetic differentiation among populations (*F*_ST_) is significantly lower in *A. anatina* (calculated for sites with at least five individuals; Wilcoxon rank‐sum test: *P* < 0.0001). (D) The average kinship coefficient within populations is significantly lower in *A. anatina* (calculated for all sites with *n* > 1; Wilcoxon rank‐sum test: *P* < 0.01). (E) The ROH‐based inbreeding coefficient (FRoH) is significantly lower in *A. anatina* (calculated for all sites; Wilcoxon rank‐sum test: *P* < 0.0001). (F) The inbreeding coefficient (*F*_IS_) does not differ between *A. anatina* and *A. cygnea* (calculated for sites with at least five individuals; Wilcoxon rank‐sum test: *P* > 0.05). (G) Effective population size (*N*e) is significantly larger in *A. anatina* (calculated for sites with at least ten individuals; Wilcoxon rank‐sum test: *P* < 0.01).

Due to the low number of individuals collected for *A. exulcerata* and *Anodonta* sp., further inferences regarding genetic EBVs in these species were not made. Sufficient sample sizes and geographic coverage were, however, obtained for *A. anatina* and *A. cygnea* , allowing more detailed comparisons. A striking finding was the substantial differences observed in genetic EBV values between the two species, which differ primarily in their reproductive strategies— *A. anatina* being an obligate outcrosser and *A. cygnea* likely a facultative selfer. In terms of genetic diversity, this change in reproductive mode was associated with a 10‐fold reduction in nucleotide diversity and an 11‐fold reduction in observed heterozygosity, both of which were highly significant (Figure 6A,B). Regarding genetic differentiation, *A. cygnea* exhibited, on average, three times as high genetic differentiation (*F*_ST_) between locations and four times as high relatedness within each location (Figure 6C,D). In terms of inbreeding, the shift in reproductive mode increased individual inbreeding (FRoH) by a factor of 36, while the population inbreeding coefficients (*F*_IS_) were not significantly differentiated among species (Figure 6E,F). Finally, effective population size was reduced by approximately threefold in *A. cygnea* (Figure 6G).

## 4 Discussion

Freshwater mussels play a crucial role in aquatic ecosystems but are among the most endangered aquatic invertebrates. Despite their ecological importance, limited genomic data hinder conservation efforts. In this study, we generated draft genomes for *A. cygnea* and *A. anatina* and resequenced 421 individuals across Switzerland. Our findings reveal key genetic insights, including the discovery of a putative undescribed *Anodonta* species south of the Alps, strong genetic structuring of *A. anatina* by catchment area, and reduced genetic diversity in small pond populations, indicating higher vulnerability. Notably, we show that a shift in the reproductive mode of *A. cygnea* , most likely facultative selfing, drastically lowers genetic diversity and effective population sizes, increasing extinction risk. These results highlight the need to integrate reproductive strategies into conservation planning and emphasise the importance of habitat connectivity and genetic monitoring for freshwater mussel conservation.

### 4.1 New Genomic Resources for European Unionids

While the essential role of reference genomes in biodiversity conservation has been well established (Formenti et al. 2022; Theissinger et al. 2023), genomic resources for freshwater mussels remain scarce, with only eight published genomes currently available (Renaut et al. 2018; Rogers et al. 2021; Smith 2021; Gomes‐dos‐Santos, Froufe, Machado, Lajtner, et al. 2023; Gomes‐dos‐Santos, Lopes‐Lima, Machado, Forest, et al. 2023; Gomes‐dos‐Santos, Lopes‐Lima, Machado, Teixeira, et al. 2023; Bai et al. 2024; Ma et al. 2024) (Table S3). By providing two additional genome assemblies, our study contributes to filling this gap. Despite considerable efforts, the two draft genomes differ in their quality, mainly caused by the difference in sequencing coverage that was achievable. The fragmented nature of the *A. anatina* reference genome presents some limitations for downstream analyses. Fragmentation can reduce the resolution of analyses that depend on contiguity, such as synteny comparisons or fine‐scale genome scans, and may slightly lower the power to detect divergence. However, it is unlikely to introduce false signals of genetic differentiation. Major population structure, species divergence and phylogenetic relationships—as assessed here using genome‐wide markers—are generally robust to assembly fragmentation, particularly when based on large numbers of SNPs derived from across the genome. Interestingly, the assembly quality metrics of the *A. cygnea* genome were comparable to other published unionid genomes, despite lower sequencing coverage (Table S3). This suggests that high‐quality assemblies can be obtained with a modest PacBio HiFi coverage, making draft genome sequencing more cost‐effective. Furthermore, comparisons with previously published genomes indicate that achieving chromosome‐scale assemblies requires additional technologies (e.g., Hi‐C) and that solely increasing PacBio coverage has only a marginal impact on assembly contiguity. Finally, our findings suggest that the Mollusca Benchmarking Universal Single‐Copy Orthologs (BUSCO) database may not be fully appropriate for Unionids, which likely exhibit true gene losses. Even the chromosome‐scale reference genome of *Sinosolenaia oleivora* had a BUSCO completeness score of only 88.6% (Ma et al. 2024), highlighting the need to revise the Mollusca database of single‐copy orthologs, as previously suggested for Patellids (Halstead‐Nussloch et al. 2024).

### 4.2 A Previously Uncharacterised *Anodonta* Taxon South of the Alps

Our results reveal cryptic diversity in the Ticino catchment area, south of the Alps, where we identified a population of *Anodonta* sp., representing a putative undescribed species. The potential existence of cryptic species within *A. anatina* has been previously suggested, where large‐scale European studies have documented five divergent mitochondrial lineages, of which at least three had corresponding nuclear divergence, supporting their recognition as distinct evolutionary significant units (ESUs) (Froufe et al. 2014, 2017; Lopes‐Lima et al. 2016). Our findings confirm that one of these ESUs, the Ebro & Italy clade, is likely a separate species based on significant nuclear divergence highlighted here. Interestingly, we also uncovered genomic signatures of hybridisation between *A. anatina* and *Anodonta* sp., highlighting that these genomes are still able to recombine, similar to *A. cygnea* and *A. exulcerata*. Although we did not conduct a detailed morphological analysis, it is reasonable to assume that *A. anatina* and *Anodonta* sp. lack diagnostic morphological differences, given their recent evolutionary divergence and the well‐documented morphological plasticity of this genus (e.g., lack of diagnostic morphological characters between *A. anatina* and *A. exulcerata* ) (Riccardi et al. 2020). Finally, as our study is based on a single population with a limited number of individuals, future research should prioritise comprehensive sampling across the full geographic range of this lineage, morphological studies, species delimitation analyses as well as a formal taxonomic analysis before taxonomic revision can be proposed.

### 4.3 Genetic Essential Biodiversity Variables for *Anodonta anatina*

A key finding regarding genetic diversity in *A. anatina* is that both nucleotide diversity and heterozygosity were positively correlated with water body size, suggesting that populations inhabiting small ponds are more vulnerable than those in large lakes, due to their lower adaptive potential. A positive correlation between genome‐wide heterozygosity and geographic range was also recently found in Felidae (Meeus et al. 2025). Analysis of genetic differentiation revealed strong structuring by catchment area and isolation by distance, with most populations also being significantly differentiated from one another, emphasising the species' metapopulation structure. Because mussel dispersal primarily occurs via glochidia parasitising host fish, our findings highlight the importance of maintaining connectivity among water bodies by avoiding barriers that restrict fish movement. *A. anatina* is considered a host generalist, capable of parasitising multiple fish species, though studies suggest a preference for *Perca fluviatilis* (Huber and Geist 2019). Therefore, maintaining and enhancing fish connectivity is essential for conserving *A. anatina* , as it facilitates mussel dispersal and promotes genetic exchange among otherwise isolated populations.

Inbreeding levels were generally low, indicating genetic mixing among diverse individuals. However, estimates of the fraction of runs of homozygosity (FRoH) should be interpreted cautiously due to the fragmented nature of the draft genome and the low sequencing coverage (Silva et al. 2024). Our estimates of effective population size (*N*e) were low, ranging from 20 to 60, well below the recommended threshold of *N*e > 500 for maintaining long‐term evolutionary potential (Traill et al. 2010; Jamieson and Allendorf 2012). Most populations fell below 50, the critical short‐term threshold to avoid inbreeding depression, suggesting that *A. anatina* populations face a high extinction risk. These low *N*e values may be the result of recent population bottlenecks after the intense period of eutrophication freshwater ecosystems faced in the 1960s–1980s (Vonlanthen et al. 2012). We acknowledge that our *N*e estimates may be underestimated due to the relatively small sample sizes, and that *N*e remains a challenging metric to estimate accurately (Waples 2025). Yet, even if the *N*e of small connected lakes and ponds within each catchment were combined—assuming that other unsampled populations have similar sizes—it is unlikely that the metapopulation *N*e would reach 500. To complement genetic estimates, direct assessments of census population size (*Nᴄ*) would be valuable. As all mussels in this study were marked before release, a recapture experiment in the coming years could provide insights into *Nc* and allow comparison with *N*e estimates, as the *N*e/*Nc* ratio is increasingly recognised as an important metric in applied conservation (Waples 2024).

### 4.4 Hermaphroditism and Selfing: Reproductive Advantage With Evolutionary Costs

To date, *A. cygnea* is the only unionid species in Europe described as predominantly hermaphrodite (Lima et al. 2012). *A. exulcerata* may also exhibit hermaphroditism, as it shares with *A. cygnea* the mitochondrial H‐ORF, a genetic feature characteristic of hermaphroditic species that have lost the doubly uniparental inheritance system (Breton et al. 2011, 2018; Chase et al. 2018; Riccardi et al. 2020). However, the genetic diversity within the single *A. exulcerata* population was significantly higher than *A. cygnea* , suggesting a lack of self‐fertilisation. Similarly, while *A. anatina* has been reported as facultative hermaphrodite, self‐fertilisation has not yet been directly investigated (Hinzmann et al. 2013). The evolution of hermaphroditism has been hypothesised to be favoured in situations where mates are sparsely distributed and access to compatible gametes is limited (Ghiselin 1969; Eppley and Jesson 2008). In freshwater mussels, hermaphroditism is thought to be advantageous in low‐density populations with limited water flow, where opportunities for gamete dispersal are constrained (Bauer 1987; Stewart et al. 2013). This reproductive strategy provides an immediate benefit by ensuring reproductive success in the absence of mates (Jarne and Charlesworth 1993). However, hermaphroditism—particularly when associated with high levels of selfing—carries evolutionary costs, including rapid decline in genetic diversity, and consequently, reduced adaptive potential. Evolutionary theory predicts that obligate self‐fertilising hermaphrodites represent evolutionary dead ends, which was supported by meta‐analyses (Stebbins 1957; Takebayashi and Morrell 2001). Consistent with this prediction, obligate hermaphrodites among freshwater mussels appear to have evolved relatively recently, with gonochorism (separate sexes) representing the ancestral state (Stewart et al. 2013). One possible mechanism for mitigating extinction risk is facultative selfing, combined with occasional hybridisation with closely related species, as observed here between *A. cygnea* and *A. exulcerata* . However, given that *A. cygnea* has a much broader geographic distribution than *A. exulcerata* , such genetic exchange is likely restricted to a few localised sympatric populations. As a result, across its broader range, *A. cygnea* is likely at a greater long‐term risk of extinction than *A. anatina* , despite both species having similar pan‐European distributions.

We cannot rule out the possibility that the unusual genomic signal observed in *A. cygnea* results from facultative parthenogenesis, in which embryos develop from unfertilised eggs (Smith and Shuker 2025). In particular, terminal fusion automixis can drastically reduce heterozygosity within a single generation (Lampert 2009; Lehtonen et al. 2012), potentially explaining the genomic pattern resembling clonality observed in some *A. cygnea* populations (Figure S13). Furthermore, the presence of sperm was not specifically investigated in the ‘self‐fertilised’ individuals examined (Bloomer 1940, 1943). However, facultative parthenogenesis is less common in hermaphrodites. To minimise stress on the mussels, we did not record individual sex in this study, but literature suggests that *A. cygnea* is predominantly hermaphroditic (Lima et al. 2012). Further studies specifically designed to differentiate selfing from parthenogenesis are needed to confirm this species' reproductive mode conclusively.

### 4.5 Hybridisation and Atypical Reproductive Modes Challenge Genetic EBV Assessment

Our study highlights the complexities of using genetic EBVs for conservation, particularly in species that exhibit hybridisation and atypical reproductive strategies. We detected ongoing hybridisation between *A. cygnea* and *A. exulcerata* , with at least five strongly admixed individuals identified in a sympatric population. Notably, this *A. exulcerata* population exhibited unexpectedly high genetic diversity, exceeding even that of *A. anatina* . As only a single population was sampled, it remains unclear whether this elevated diversity is an inherent feature of the species. However, a study on mitochondrial *COI* across the entire *A. exulcerata* range found that *A. exulcerata* had higher haplotype diversity than *A. cygnea* (Froufe et al. 2017). Hybridisation appears to be a common phenomenon in freshwater mussels (Sano et al. 2022; Lopes‐Lima et al. 2024). More broadly, hybridisation is known to play a significant role in the evolution of biodiversity (Taylor and Larson 2019): for instance, by facilitating adaptation or driving speciation (Meier et al. 2017), but it can also lead to negative outcomes such as outbreeding depression (Frankham et al. 2011) or genetic swamping (Todesco et al. 2016). These dual effects complicate the interpretation of EBVs, as elevated genetic diversity in hybrid populations does not necessarily equate to long‐term stability or resilience (Quilodrán et al. 2020).

Beyond hybridisation, reproductive mode also plays a crucial role in shaping genetic EBVs. Our study provides an ideal system to investigate this, as *A. anatina* and *A. cygnea* differ primarily in their reproductive strategies while sharing similar dispersal abilities, geographic distribution and evolutionary histories (Lopes‐Lima et al. 2017). This parallel allows us to attribute the observed differences in genetic EBVs primarily to their reproductive mode. We found that the change in reproductive mode of *A. cygnea* is associated with markedly reduced genetic diversity, increased population structure and inbreeding, and lower effective population sizes. Further investigation is needed to assess whether *A. cygnea* experiences inbreeding depression or can persist with consistently low genetic diversity, and whether alternative mechanisms such as phenotypic plasticity, epigenetic regulation (Venney et al. 2023; Balard et al. 2024) and/or the purging of deleterious mutations (Arunkumar et al. 2015) are alleviating its genetic constraints. The interplay between selfing and hybridisation complicates conservation assessments. While these reproductive strategies have been extensively studied in plants, increasing evidence suggests they are also widespread among animals (Avise 2015; Sperling and Glover 2023). Defining appropriate thresholds for genetic indicators in species with atypical reproductive modes remains an open question. The recently launched COST Action *Genetic Nature Observation and Action* (https://www.cost.eu/actions/CA23121/) aims to address this challenge, specifically in the Objective 3.4 of the Working Group 3. Ultimately, our study underscores the critical need to integrate species‐specific reproductive biology into conservation assessments to ensure that biodiversity monitoring and management are both accurate and effective.

## 5 Conclusion

Our study demonstrates the power of genomics to uncover cryptic diversity, assess population structure and inform freshwater mussel conservation. We identified a putative undescribed *Anodonta* species south of the Alps, highlighting the need for accurate taxonomic and genetic assessments before conservation actions. Strong population structuring in *A. anatina* underscores the importance of maintaining hydrological connectivity, while the low genetic diversity in small pond populations signals increased vulnerability. Whole‐genome resequencing provides critical insights into genetic diversity, effective population size and inbreeding, offering a baseline and early warnings before demographic declines are evident. However, genetic indicators must be interpreted cautiously in species with complex reproductive strategies like self‐fertilisation and hybridisation. Genetic monitoring should extend beyond single time points to track trends and detect early signs of genetic erosion (Schwartz et al. 2007; Hoban et al. 2014; Leroy et al. 2018). Global conservation initiatives for freshwater mussels are currently underway (Aldridge et al. 2023) and include habitat restoration, captive breeding and reintroduction programmes (Ferreira‐Rodríguez et al. 2019; Geist et al. 2023; Sousa et al. 2023). Breeding programmes, successful in other mussel species (Geist et al. 2021, 2023), could aid restoration but must consider genetic risks such as inbreeding or outbreeding depression. Without genomic resources, translocation efforts risk being ineffective (McLennan et al. 2025). Recent initiatives, such as genetic monitoring frameworks (Hogg et al. 2025), and increased collaboration between researchers and conservation practitioners (Shaw et al. 2024) will help integrate genomics into real‐world conservation. Future priorities should include expanding genomic resources, refining genetic EBVs for species with atypical reproductive modes, and strengthening partnerships with conservation stakeholders. Applying genomic insights to management will be critical for ensuring the long‐term persistence of freshwater mussel populations and broader biodiversity.

## Author Contributions

**Ellika Faust:** formal analysis, investigation, data curation, writing – original draft, writing – review and editing, visualization. **Julie Conrads:** methodology, formal analysis, investigation, data curation, writing – original draft, writing – review and editing. **Marco Giulio:** investigation, writing – review and editing. **Claudio Ciofi:** investigation, writing – review and editing. **Chiara Natali:** investigation, writing – review and editing. **Philine G. D. Feulner:** conceptualization, writing – review and editing, supervision, funding acquisition. **Alexandra A.‐T. Weber:** conceptualization, methodology, investigation, resources, writing – original draft, writing – review and editing, supervision, project administration, funding acquisition.

## Conflicts of Interest

The authors declare no conflicts of interest.

## Supporting information


**Figure S1:** Neighbour‐Joining phylogenetic tree of the *COI* marker, including 10 *Anodonta* sp. from Locarno (Ticino) and all COI haplotypes (AA1–AA18) from the *A. anatina* European (EUR) and Italian (ITA) clades (Froufe et al. 2017).
**Figure S2:** ADMIXTURE cross‐validation analysis for *A. anatina* and *Anodonta* sp. together with admixture results for *K* = 1 to *K* = 12, separated by sampling locality.
**Figure S3:** *A. anatina* and *Anodonta* sp. ADMIXTURE results for *K* = 1 to *K* = 12, separated by catchment area.
**Figure S4:** Pairwise *F*_ST_ values among all *A. anatina* and *Anodonta* sp. populations.
**Figure S5:** Maximum‐Likelihood phylogenetic tree of *A. anatina* and *Anodonta* sp. populations, coloured by sampling locality.
**Figure S6:** Maximum‐Likelihood phylogenetic tree of *A. anatina* and *Anodonta* sp. populations, coloured by catchment area.
**Figure S7:** Runs of homozygosity in *A. anatina* and *Anodonta* sp.
**Figure S8:** Population pairwise average kinship estimates among *A. anatina* and *Anodonta* sp. populations.
**Figure S9:** Genomic PCA (PC1‐PC8) for *A. cygnea* and *A. exulcerata*.
**Figure S10:** ADMIXTURE cross‐validation analysis for *A. cygnea* and *A. exuclerata* together with admixture results for *K* = 1 to *K* = 6, separated by sampling locality.
**Figure S11:** ADMIXTURE cross‐validation analysis for *A. cygnea* (excluding hybrids) together with admixture results for *K* = 1 to *K* = 10, separated by sampling locality.
**Figure S12:** Genomic PCA (PC1‐PC8) for *A. cygnea* (excluding hybrids).
**Figure S13:** Maximum‐Likelihood phylogenetic tree of *A. cygnea*, coloured by sampling locality.
**Figure S14:** Pairwise *F*_ST_ values among all *A. cygnea* and *A. exulcerata* populations.
**Figure S15:** Population pairwise average kinship estimates among *A. cygnea* populations.
**Figure S16:** Absence of strong association between genetic indicators (observed heterozygosity, inbreeding coefficient, nucleotide diversity, fraction of runs of homozygosity, effective population size) and waterbody size in *A. cygnea*.
**Figure S17:** Absence of isolation by distance in *A. cygnea*.

**Table S1:** Metadata including individual codes and ENA accession numbers of the 421 *Anodonta* individuals collected in this study.
**Table S2:** Filtering parameters and number of variants at each filtering step for the three VCF files used in this study.
**Table S3:** Statistics of the *Anodonta cygnea* and *Anodonta anatina* draft genomes generated in this study and comparison with published genomes of freshwater mussels (order Unionida).
**Table S4:** *Anodonta anatina* mitochondrial lineage and GenBank accession numbers of the COI sequences retrieved from Froufe et al. (2017), used for the phylogenetic tree displayed in Figure S1.
**Table S5:** Pairwise *F*_ST_ values (Hudson estimator) among *A. anatina* populations having five individuals or more.
**Table S6:** Pairwise individual kinship estimates among all *A. anatina* and *Anodonta* sp. individuals.
**Table S7:** Pairwise *F*_ST_ values (Hudson estimator) among *A. cygnea* and *A. exulcerata* populations having five individuals per species or more.
**Table S8:** Pairwise individual kinship estimates among all *A. cygnea* and *A. exulcerata* individuals.

## Data Availability

The raw Illumina resequencing data of the 421 *Anodonta* individuals have been submitted to the European Nucleotide Archive (ENA) and can be accessed under the BioProject ID PRJEB86155 (accession numbers ERS23745809‐ERS23746229). The raw PacBio sequencing data of *A. cygnea* have been submitted to the ENA and can be accessed under the BioProject ID PRJEB77385. The assembled draft genomes of *A. cygnea*, *A. anatina*, the filtered VCF files, the COI alignment and all scripts used in this study are available on the Eawag database ERIC Open (https://doi.org/10.25678/000DY2) and on GitHub https://github.com/ellikafaust/anodonta. Mussel occurrence data have been reported to the Swiss Center for the Cartography of the Fauna (info fauna) and have been linked with the Global Biodiversity Information Facility (GBIF) and the National database of genetic diversity from populations of wild species (GenDiB—project ID: gendib00000143) available at https://doi.org/10.15468/p3yn5j.
