# Supplementary material for: Beyond Genetic Indicators: How Reproductive Mode and Hybridisation Challenge Freshwater Mussel Conservation
Source: Mol Ecol. 2025 Aug 11;34(21):e70066. doi: 10.1111/mec.70066 (PMC12573723; doi:10.1111/mec.70066)

Beyond genetic indicators: how reproductive mode and hybridization challenge freshwater mussel conservation

Ellika Faust*, Julie Conrads*, Marco Giulio, Claudio Ciofi, Chiara Natali, Philine G.D. Feulner, Alexandra A.-T. Weber

*Equal contribution

Supplementary Material

Table of contents:

Figure S1: Neighbour-Joining phylogenetic tree of the *COI* marker, including 10 *Anodonta* sp. from Locarno (Ticino) and all *COI* haplotypes (AA1–AA18) from the *A. anatina* European (EUR) and Italian (ITA) clades (Froufe et al., 2017) (p. 2)
Figure S2: ADMIXTURE cross-validation analysis for *A. anatina* and *Anodonta* sp. together with admixture results for K=1 to K=12, separated by sampling locality. (p. 3)
Figure S3: *A. anatina* and *Anodonta* sp. ADMIXTURE results for K=1 to K=12, separated by catchment area. (p. 4)
Figure S4: Pairwise F_{ST} values among all *A. anatina* and *Anodonta* sp. populations. (p. 5)
Figure S5: Maximum-Likelihood phylogenetic tree of *A. anatina* and *Anodonta* sp. populations, coloured by sampling locality. (p. 6)
Figure S6: Maximum-Likelihood phylogenetic tree of *A. anatina* and *Anodonta* sp. populations, coloured by catchment area. (p. 7)
Figure S7: Runs of homozygosity in *A. anatina* and *Anodonta* sp. (p. 7)
Figure S8: Population pairwise average kinship estimates among *A. anatina* and *Anodonta* sp. populations. (p. 8)
Figure S9: Genomic PCA (PC1-PC8) for *A. cygnea* and *A. exulcerata.* (p. 9)
Figure S10: ADMIXTURE cross-validation analysis for *A. cygnea* and *A. exuclerata* together with admixture results for K=1 to K=6, separated by sampling locality. (p. 10)
Figure S11: ADMIXTURE cross-validation analysis for *A. cygnea* (excluding hybrids) together with admixture results for K=1 to K=10, separated by sampling locality. (p. 11)
Figure S12: Genomic PCA (PC1-PC8) for *A. cygnea* (excluding hybrids). (p. 12)
Figure S13: Maximum-Likelihood phylogenetic tree of *A. cygnea*, coloured by sampling locality. (p. 13)
Figure S14: Pairwise F_{ST} values among all *A. cygnea* and *A. exulcerata* populations. (p. 14)
Figure S15: Population pairwise average kinship estimates among *A. cygnea* populations. (p. 15)
Figure S16: Absence of strong association between genetic indicators (observed heterozygosity, inbreeding coefficient, nucleotide diversity, fraction of runs of homozygosity, effective population size) and waterbody size in *A. cygnea.* (p. 16)
Figure S17: Absence of isolation by distance in *A. cygnea.* (p. 17)

Figure S1: Neighbour-Joining phylogenetic tree of the *COI* marker, including 10 *Anodonta* sp. from Locarno (Ticino) and all *COI* haplotypes (AA1–AA18) from the *A. anatina* European (EUR) and Italian (ITA) clades (Froufe et al., 2017). Based on 100 bootstrap replicates; nodes with ≥90% bootstrap support are shown.

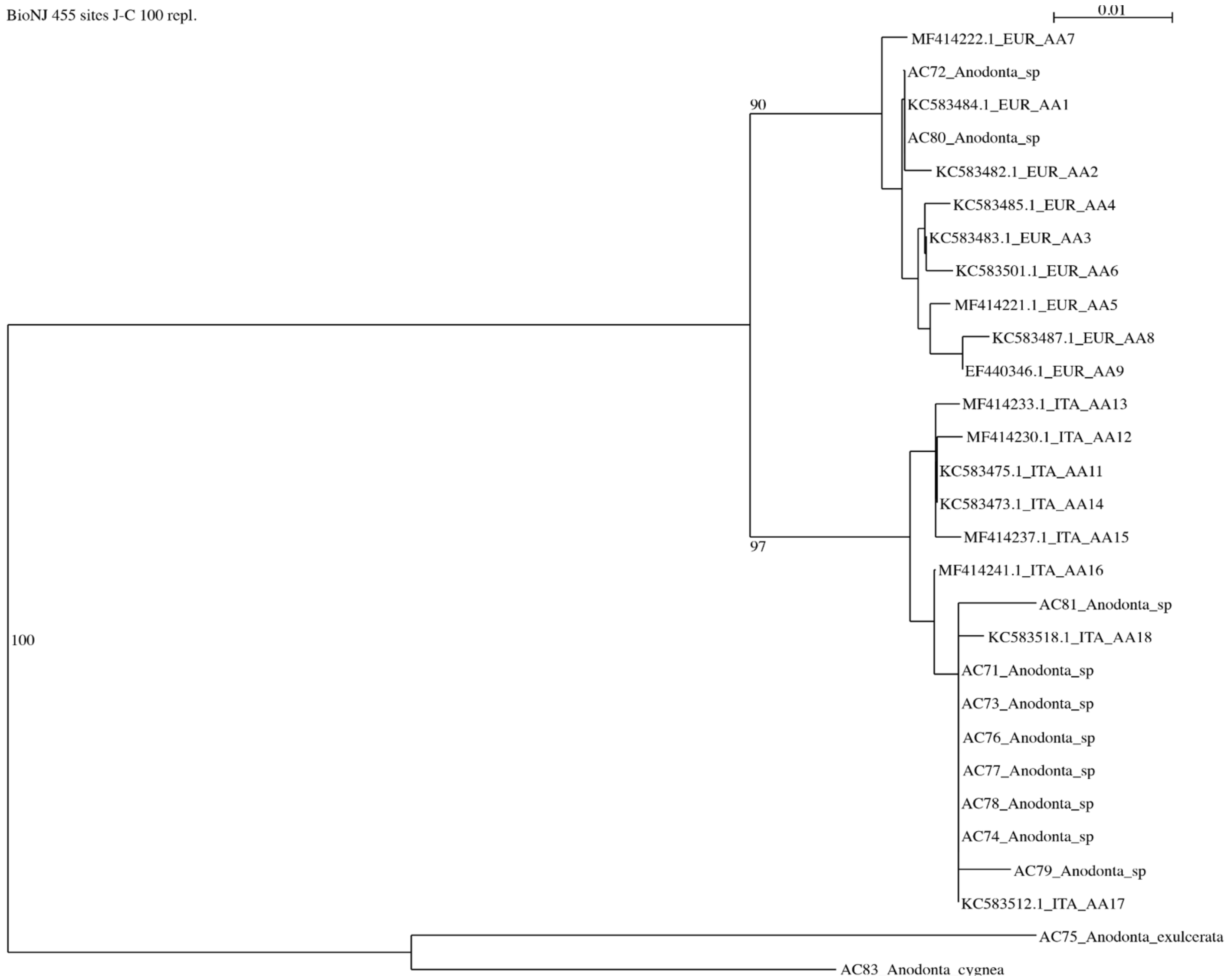

Figure S2: ADMIXTURE cross-validation analysis for *A. anatina* and *Anodonta* sp. together with admixture results for K=1 to K=12, separated by sampling locality.

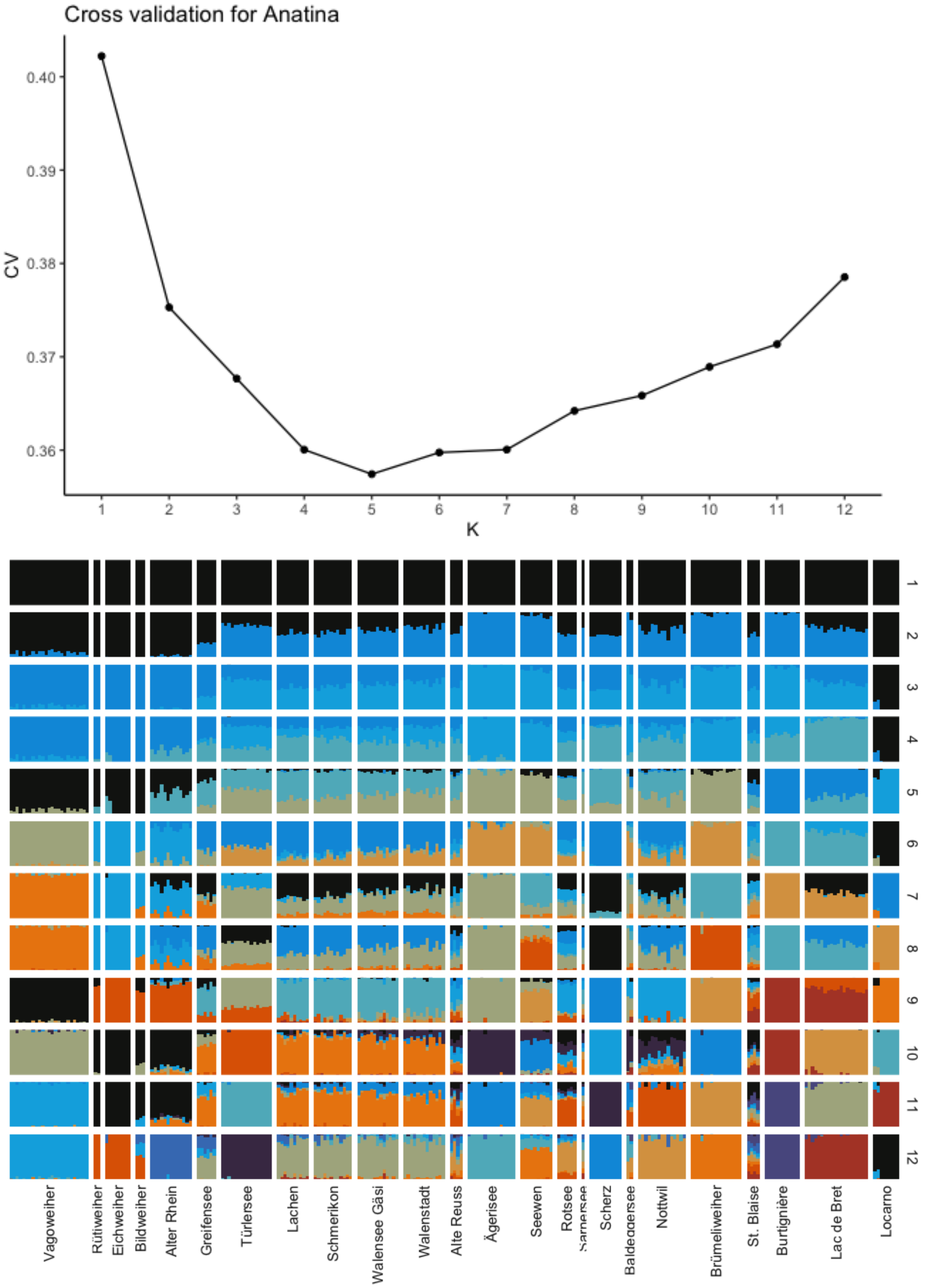

Figure S3: *A. anatina* and *Anodonta* sp. ADMIXTURE results for K=1 to K=12, separated by catchment area.

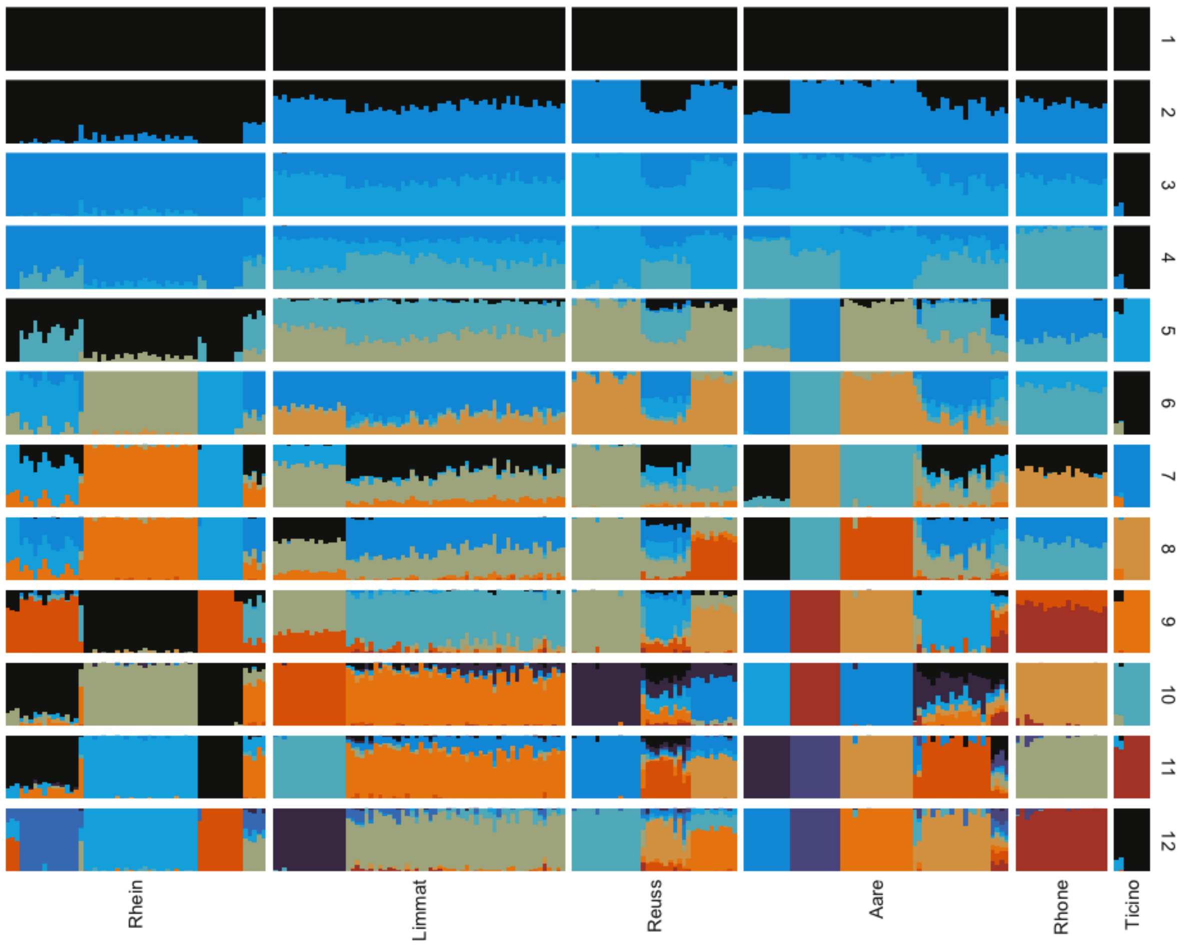

Figure S4: Pairwise F_{ST} values among all *A. anatina* and *Anodonta* sp. populations.

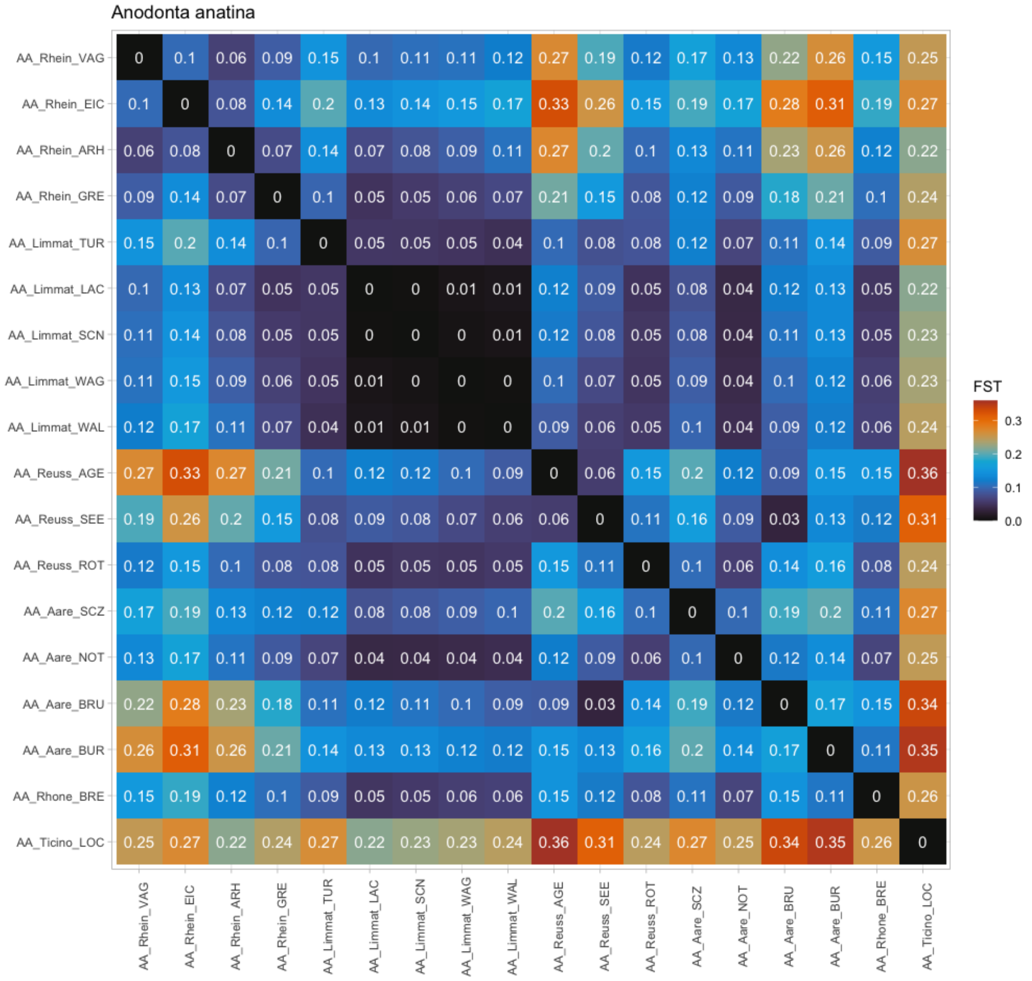

Figure S5: Unrooted Maximum-Likelihood phylogenetic tree of *A. anatina* and *Anodonta* sp. populations, coloured by sampling locality.

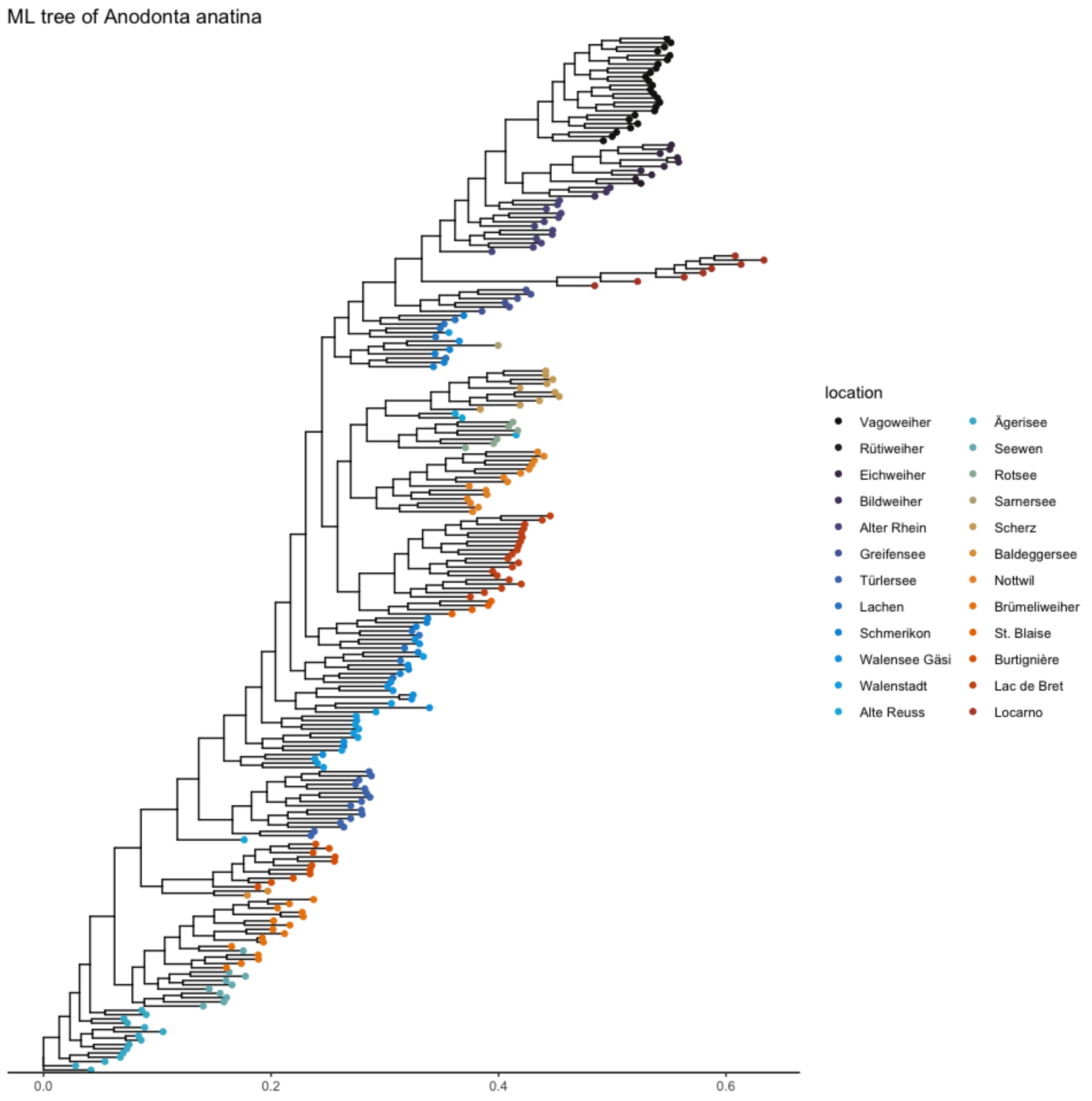

Figure S6: Unrooted Maximum-Likelihood phylogenetic tree of *A. anatina* and *Anodonta* sp. populations, coloured by catchment area.

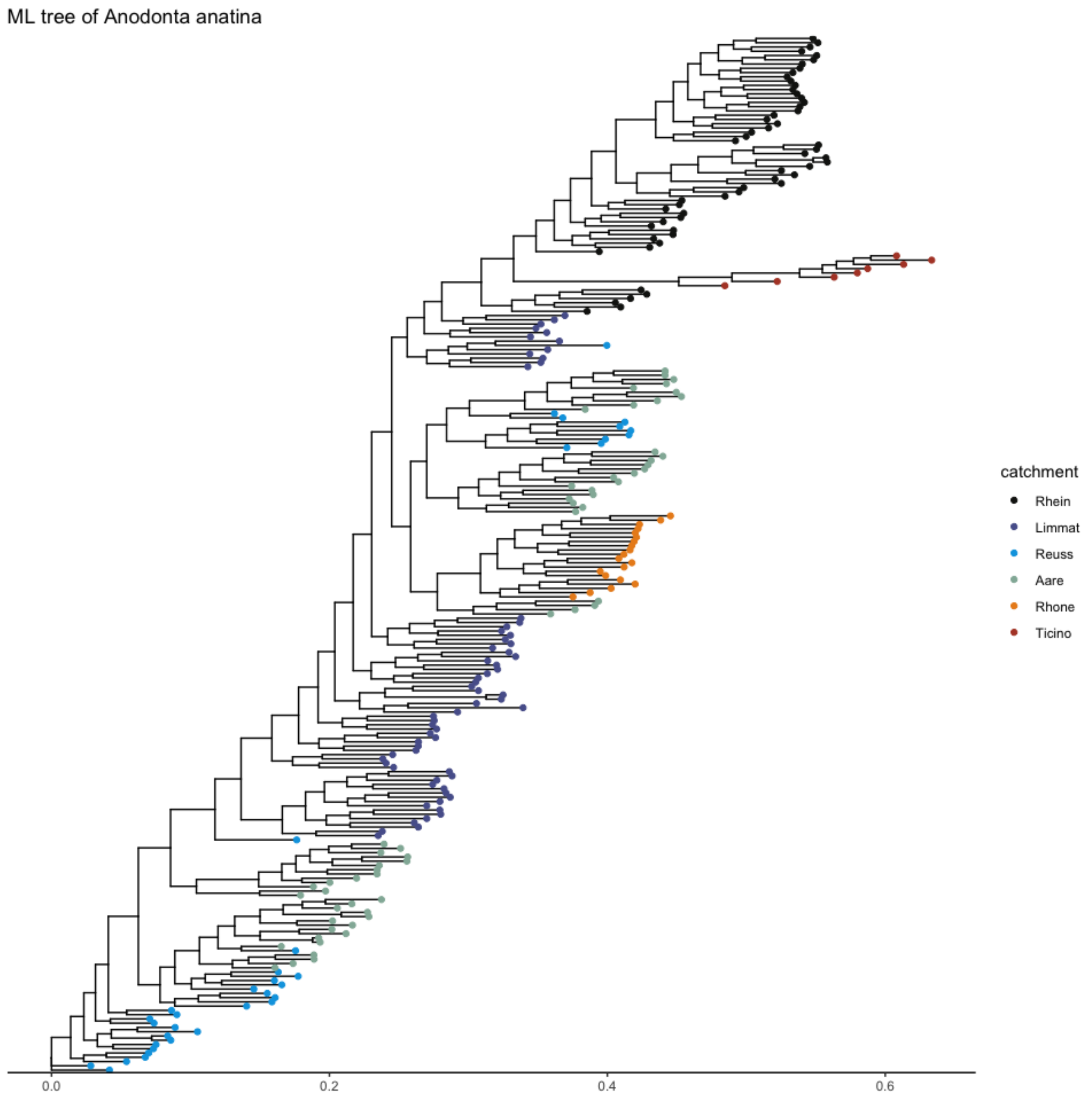

Figure S7: Runs of homozygosity in *A. anatina* and *Anodonta* sp.

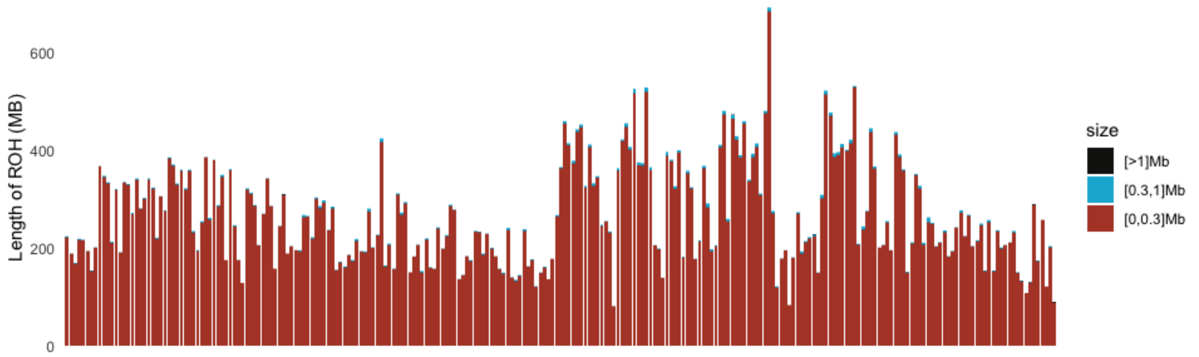

Figure S8: Population pairwise average kinship estimates among *A. anatina* and *Anodonta* sp. populations.

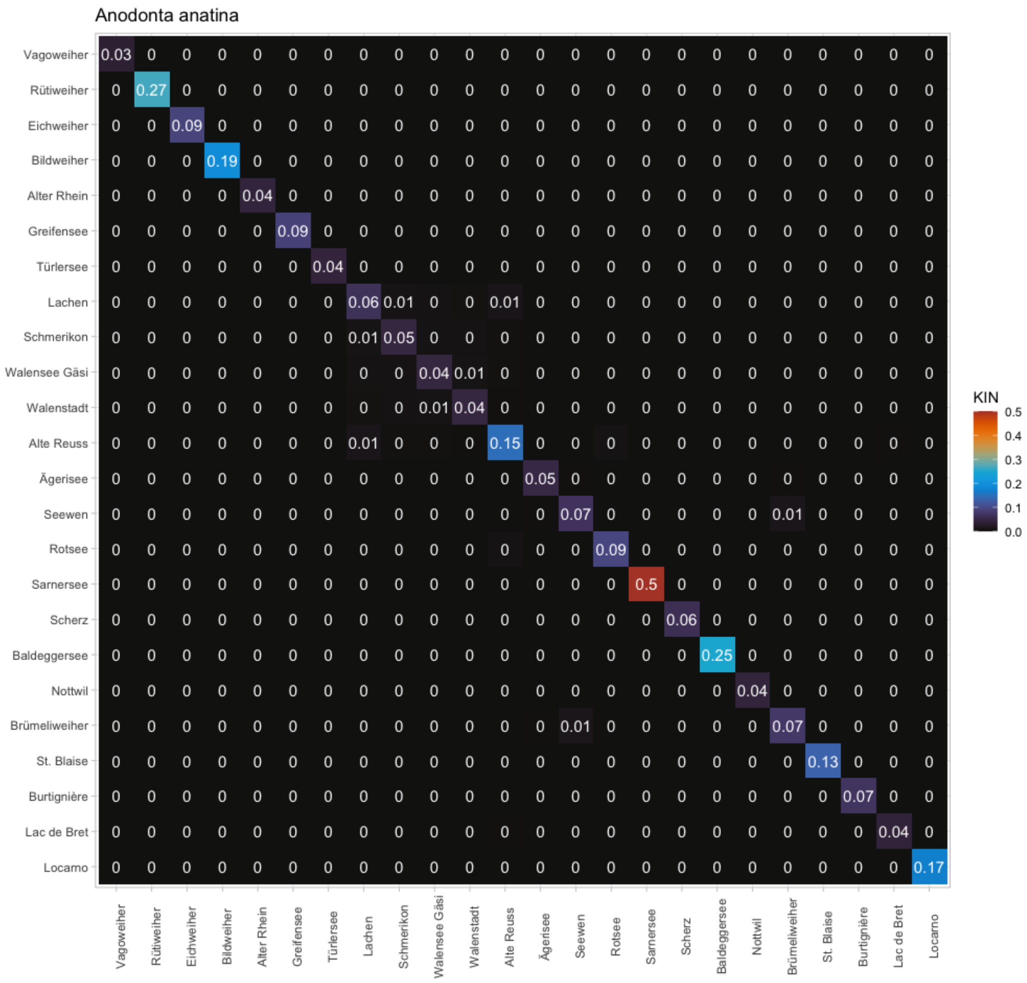

Figure S9: Genomic PCA (PC1-PC8) for *A. cygnea* and *A. exulcerata.*

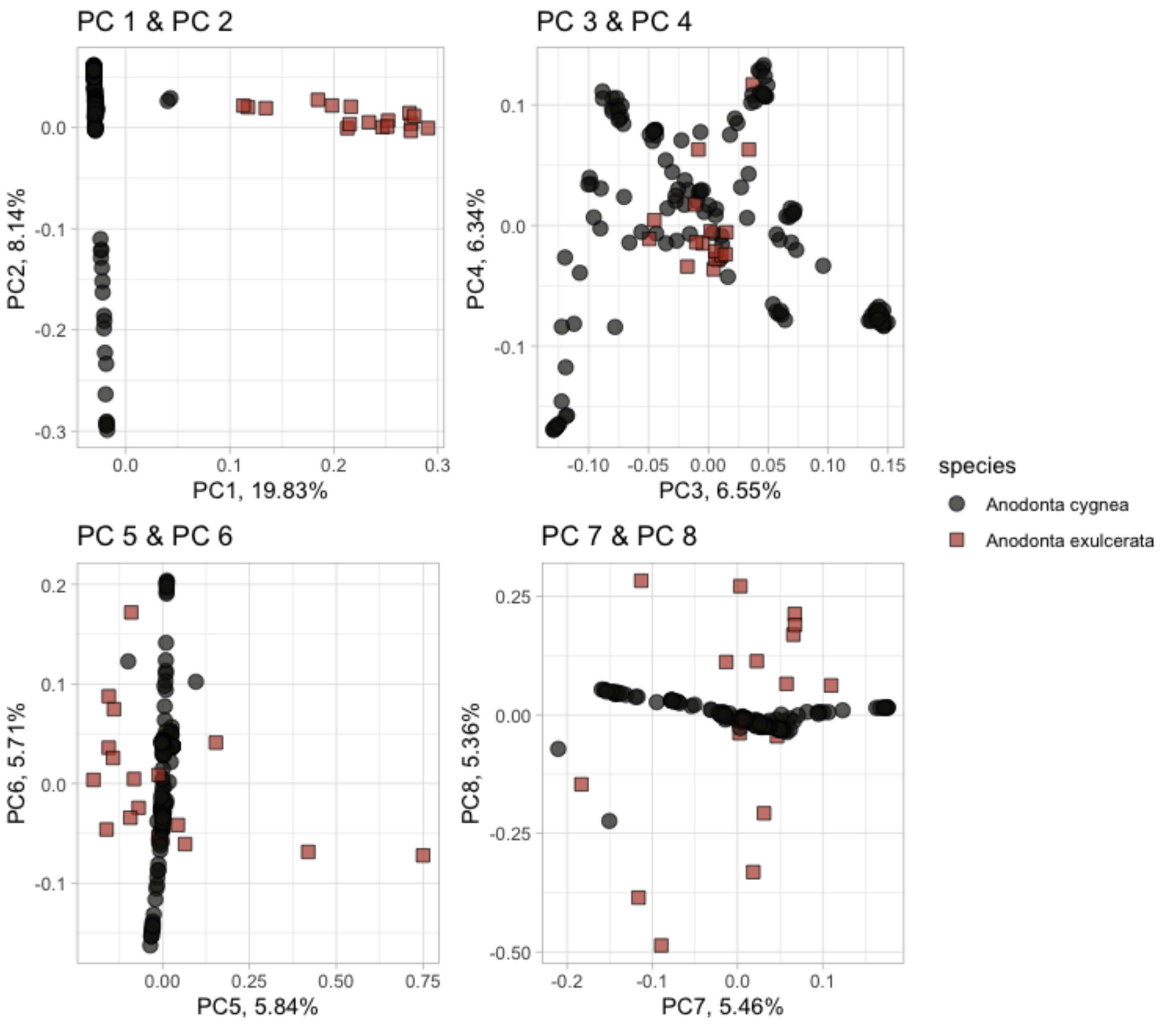

Figure S10: ADMIXTURE cross-validation analysis for *A. cygnea* and *A. exuclerata* together with admixture results for K=1 to K=6, separated by sampling locality.

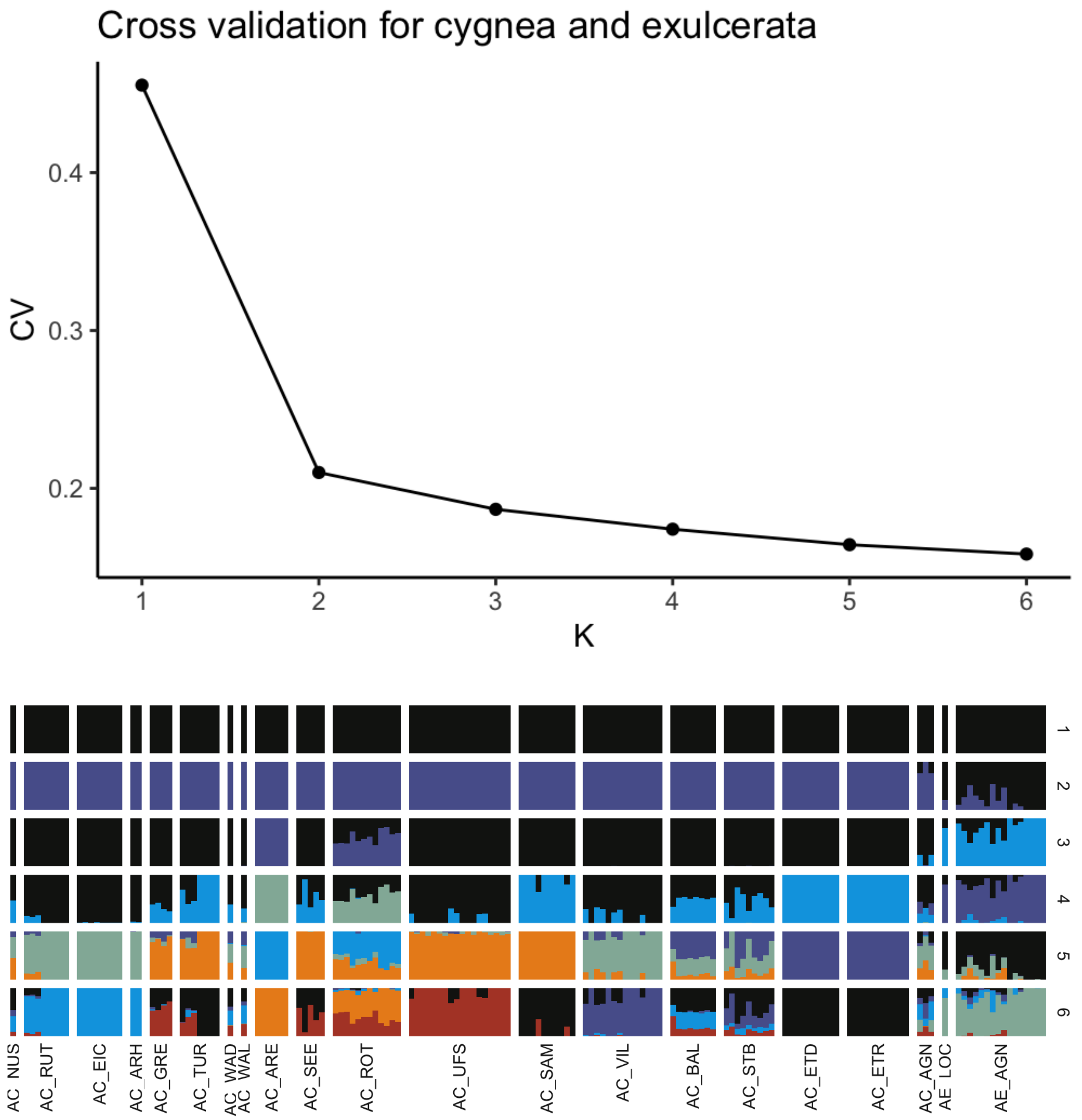

Figure S11: ADMIXTURE cross-validation analysis for *A. cygnea* (excluding hybrids) together with admixture results for K=1 to K=10, separated by sampling locality.

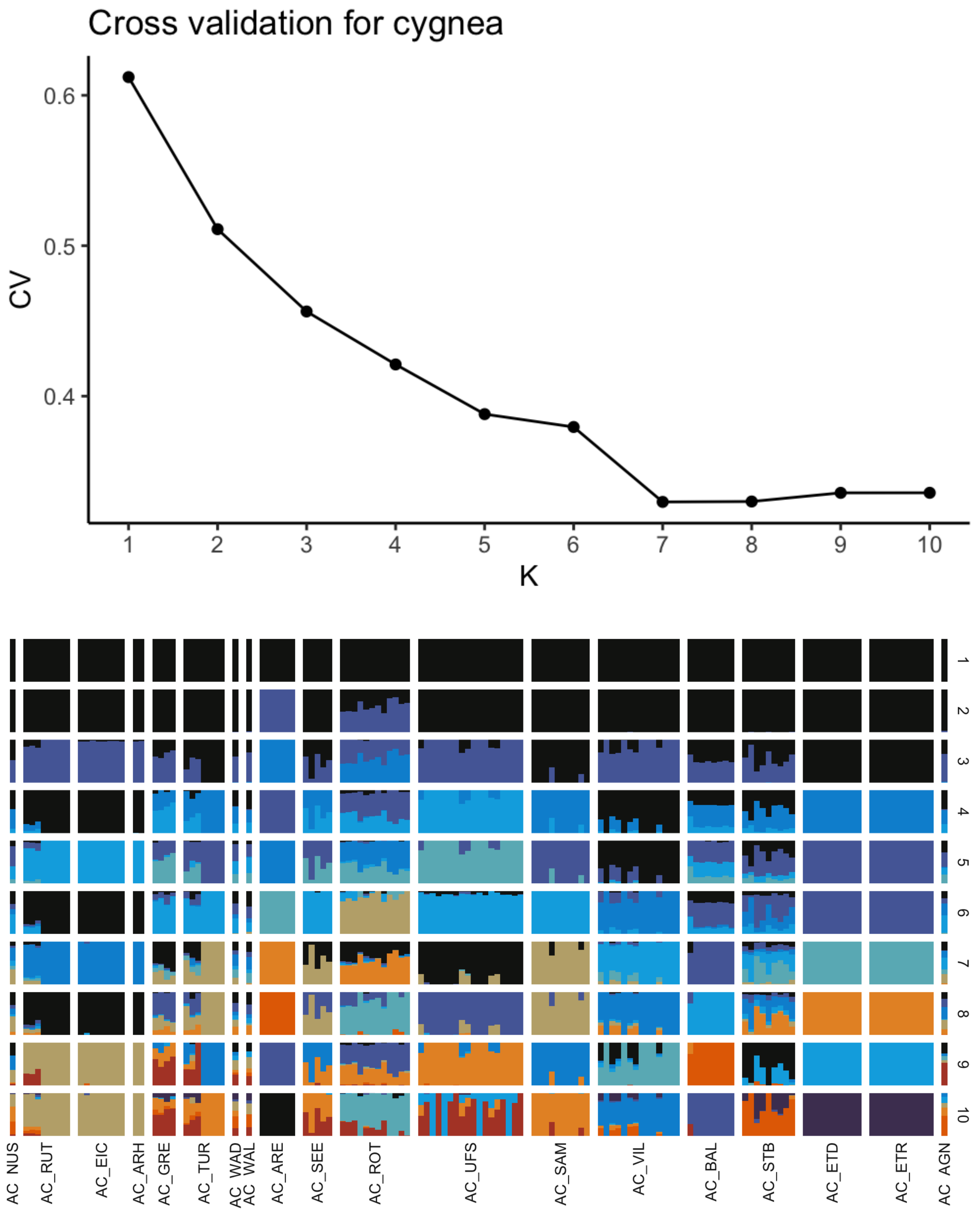

Figure S12: Genomic PCA (PC1-PC8) for *A. cygnea* (excluding hybrids).

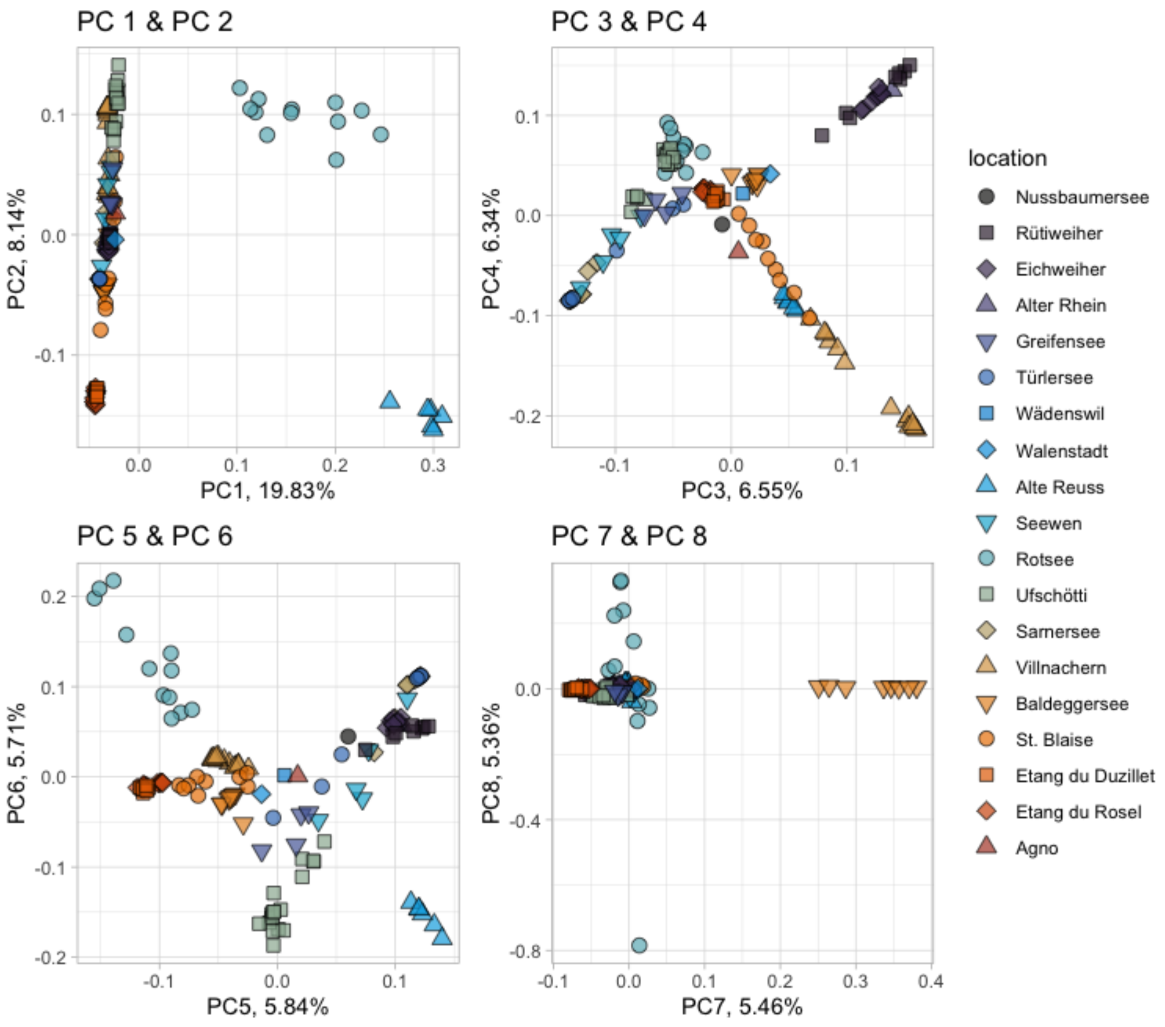

Figure S13: Unrooted Maximum-Likelihood phylogenetic tree of *A. cygnea*, coloured by sampling locality.

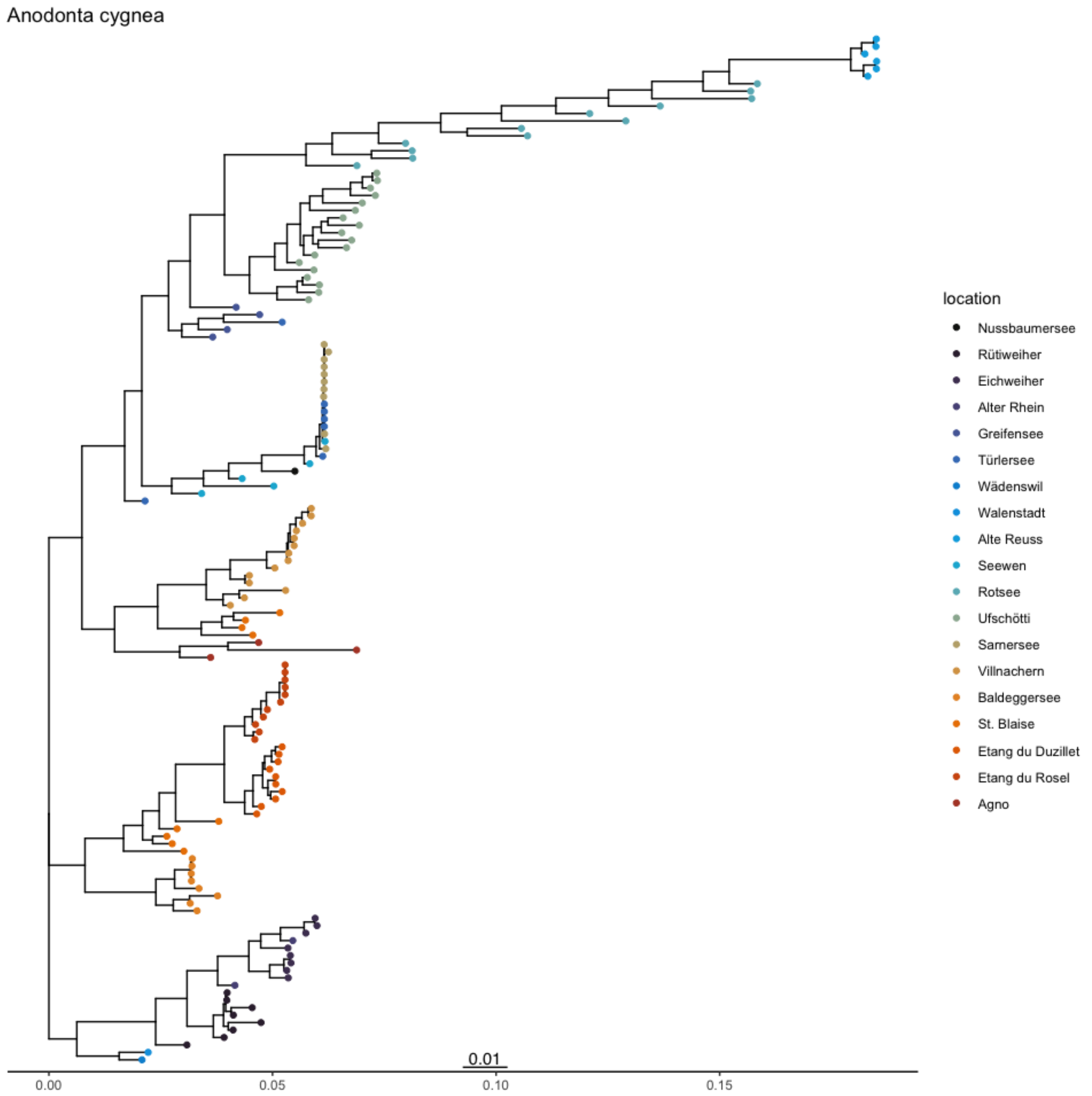

Figure S14: Pairwise F_{ST} values among all *A. cygnea* and *A. exulcerata* populations.

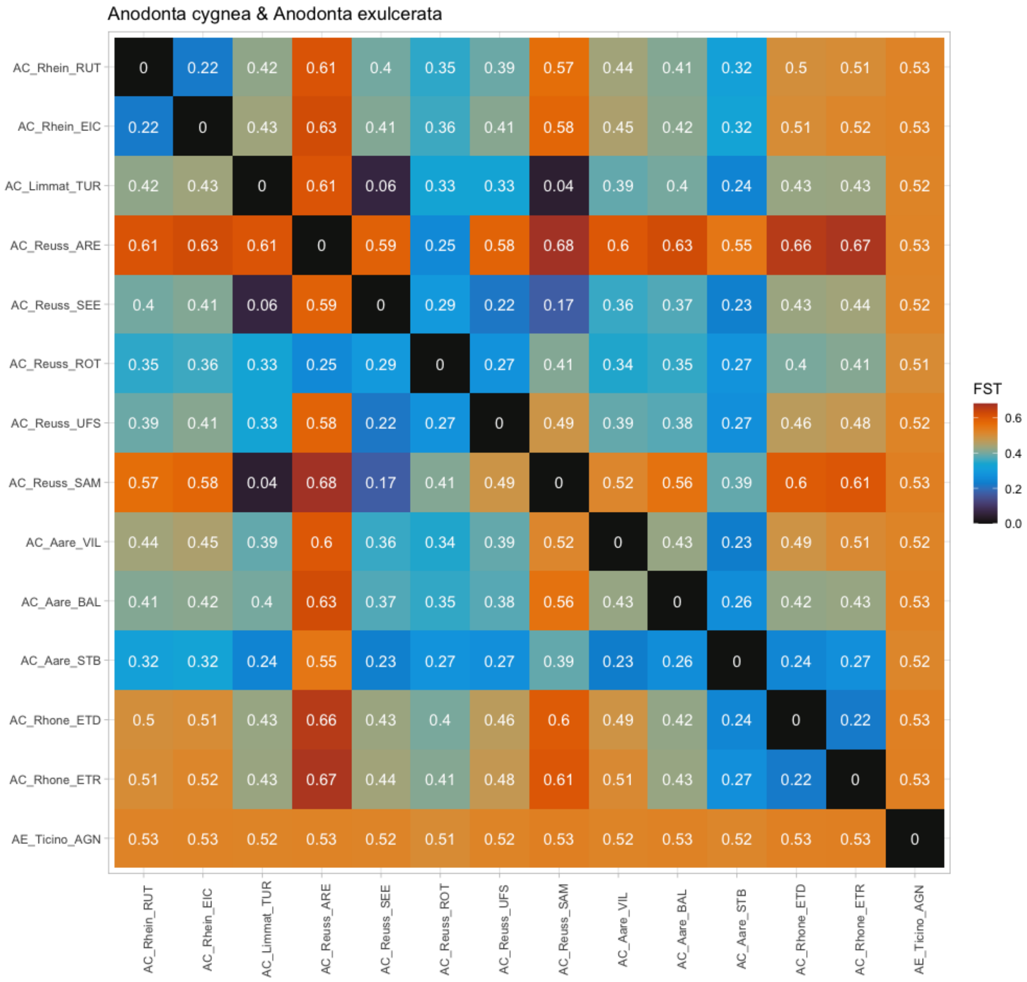

Figure S15: Population pairwise average kinship estimates among *A. cygnea* populations.

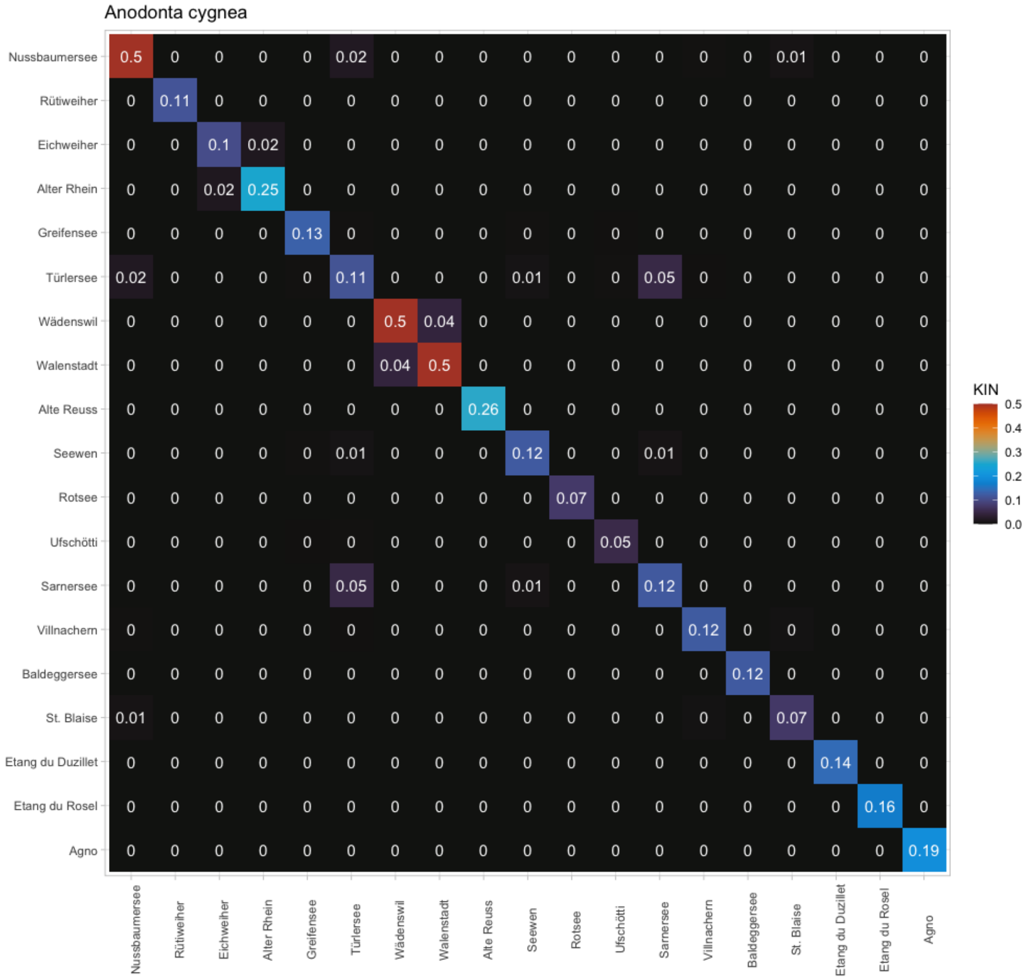

Figure S16: Absence of strong association between genetic indicators (observed heterozygosity, inbreeding coefficient, nucleotide diversity, fraction of runs of homozygosity, effective population size) and waterbody size in *A. cygnea.*

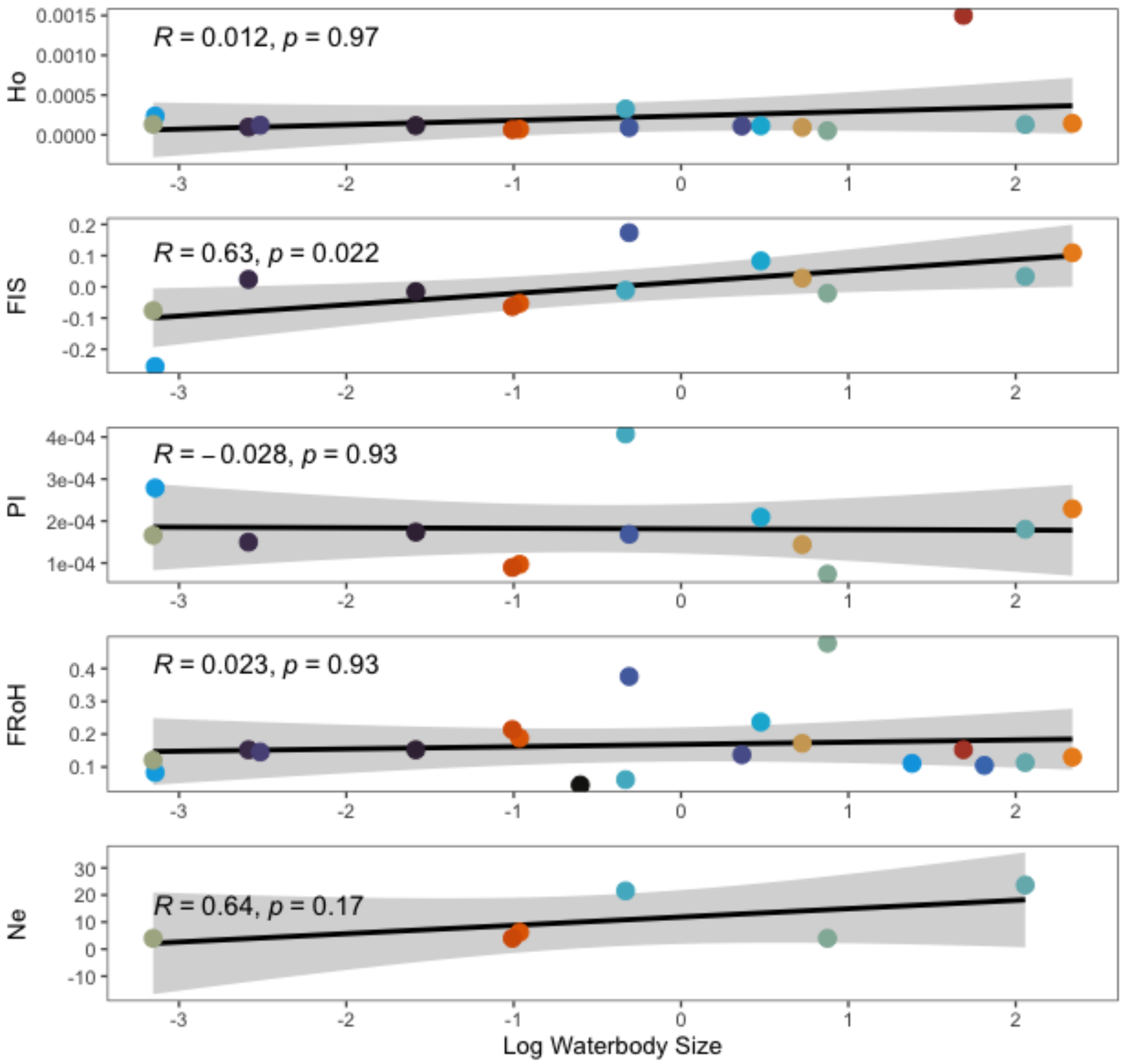

Figure S17: Absence of isolation by distance in *A. cygnea.*

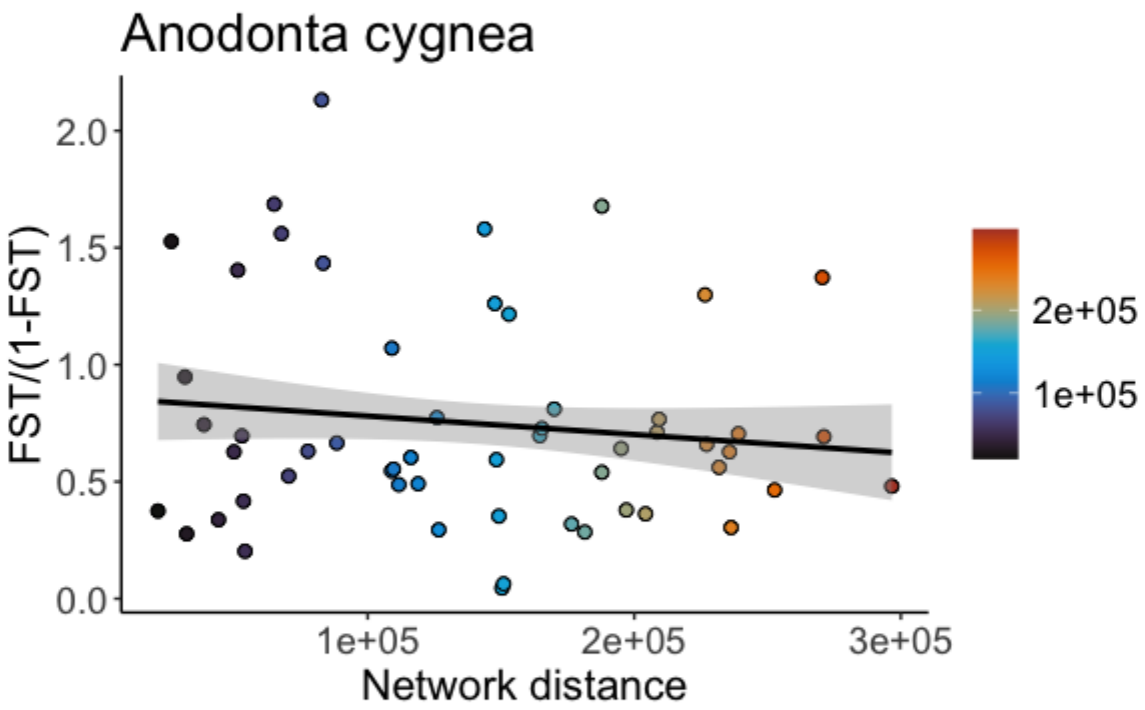

Supplement: Supplementary file 1 — Figure S1: Neighbour‐Joining phylogenetic tree of the COI marker, including 10 Anodonta sp. from Locarno (Ticino) and all COI haplotypes (AA1–AA18) from the A. anatina European (EUR) and Italian (ITA) clades (Froufe et al. 2017). Figure S2: ADMIXTURE cross‐validation analysis for A. anatina and Anodonta sp. together with admixture results for K = 1 to K = 12, separated by sampling locality. Figure S3: A. anatina and Anodonta sp. ADMIXTURE results for K = 1 to K = 12, separated by catchment area. Figure S4: Pairwise F ST values among all A. anatina and Anodonta sp. populations. Figure S5: Maximum‐Likelihood phylogenetic tree of A. anatina and Anodonta sp. populations, coloured by sampling locality. Figure S6: Maximum‐Likelihood phylogenetic tree of A. anatina and Anodonta sp. populations, coloured by catchment area. Figure S7: Runs of homozygosity in A. anatina and Anodonta sp. Figure S8: Population pairwise average kinship estimates among A. anatina and Anodonta sp. populations. Figure S9: Genomic PCA (PC1‐PC8) for A. cygnea and A. exulcerata. Figure S10: ADMIXTURE cross‐validation analysis for A. cygnea and A. exuclerata together with admixture results for K = 1 to K = 6, separated by sampling locality. Figure S11: ADMIXTURE cross‐validation analysis for A. cygnea (excluding hybrids) together with admixture results for K = 1 to K = 10, separated by sampling locality. Figure S12: Genomic PCA (PC1‐PC8) for A. cygnea (excluding hybrids). Figure S13: Maximum‐Likelihood phylogenetic tree of A. cygnea, coloured by sampling locality. Figure S14: Pairwise F ST values among all A. cygnea and A. exulcerata populations. Figure S15: Population pairwise average kinship estimates among A. cygnea populations. Figure S16: Absence of strong association between genetic indicators (observed heterozygosity, inbreeding coefficient, nucleotide diversity, fraction of runs of homozygosity, effective population size) and waterbody size in A. cygnea. Figure S17: Absence of isolation by dist [file MEC-34-e70066-s001.pdf]
